# Alpha-Lipoic Acid and Biotin in Neurodegenerative Diseases: Convergent Mechanistic Insights from Preclinical Models to Clinical Perspectives

**DOI:** 10.3390/neurolint18040064

**Published:** 2026-03-26

**Authors:** Asdrubal Aguilera-Méndez, Karel Aguilera-Manuel, Alfredo Saavedra-Molina, Patricia Ríos-Chávez, Santiago Villafaña, Renato Nieto-Aguilar, Daniel Godínez-Hernández, Daniel Ortega-Cuellar, Zoraya Palomera-Sanchez, Marcia Gauthereau-Torres

**Affiliations:** 1 Institute of Chemical-Biological Research, Universidad Michoacana de San Nicolás de Hidalgo, Morelia 58030, Mexico; francisco.saavedra@umich.mx (A.S.-M.); daniel.godinez@umich.mx (D.G.-H.); 2 Faculty of Medicine and Biological Sciences, “Dr. Ignacio Chávez”, Universidad Michoacana de San Nicolás de Hidalgo, Morelia 58020, Mexico; 2215174e@umich.mx; 3 Faculty of Biology, Universidad Michoacana de San Nicolás de Hidalgo, Morelia 58030, Mexico; patricia.rios@umich.mx; 4 Graduate Studies and Research Section, Higher School of Medicine, Instituto Politécnico Nacional, Mexico City 11340, Mexico; svillafana@ipn.mx; 5 Division of Graduate Studies and Research, Faculty of Dentistry, Universidad Michoacana de San Nicolás de Hidalgo, Morelia 58337, Mexico; rnieto@umich.mx; 6 Experimental Nutrition Laboratory, National Institute of Pediatrics, Ministry of Health, Mexico City 04530, Mexico; lcamachoc@pediatria.gob.mx; 7 Faculty of Veterinary Medicine and Animal Science, Universidad Michoacana de San Nicolás de Hidalgo, Morelia 58130, Mexico; zoraya.palomera@umich.mx; 8 Division of Graduate Studies, Faculty of Medicine and Biological Sciences, “Dr. Ignacio Chávez”, Universidad Michoacana de San Nicolás de Hidalgo, Morelia 58020, Mexico; marcia.gauthereau@umich.mx

**Keywords:** α-lipoic acid, biotin, neurodegenerative disease

## Abstract

Background: Neurodegenerative diseases, including Alzheimer’s disease, Parkinson’s disease, multiple sclerosis, and amyotrophic lateral sclerosis, represent a major global health burden and share convergent pathogenic mechanisms, such as mitochondrial dysfunction, oxidative stress, neuroinflammation, calcium imbalance, and neuronal loss. Despite advances in symptomatic management, effective disease-modifying therapies remain limited. Objectives: This review aims to critically synthesize mechanistic, preclinical, and clinical evidence on α-lipoic acid and biotin as candidate neuroprotective agents in neurodegenerative diseases, with emphasis on shared signaling pathways, therapeutic potential, generally favorable safety profiles, and translational limitations. Methods: A narrative and integrative review was conducted, encompassing mechanistic studies, preclinical experimental models, and clinical trials and observational studies evaluating ALA and biotin in neurodegenerative diseases. The evidence was qualitatively analyzed with attention to biological plausibility, consistency across models, and clinical relevance. Results: ALA and biotin modulate key cellular pathways implicated in neurodegeneration, including mitochondrial metabolism, redox homeostasis, inflammatory signaling, and neurovascular function. Preclinical studies consistently report beneficial effects on mitochondrial efficiency, oxidative stress, and neuroinflammatory markers. In contrast, clinical evidence remains heterogeneous, with more extensive evaluation of biotin in progressive multiple sclerosis and more limited or exploratory findings for ALA across neurodegenerative disorders. Conclusions: ALA and biotin exhibit mechanistic convergence across pathways relevant to neurodegeneration and generally favorable safety profiles. Although current evidence supports their biological plausibility as adjunctive or exploratory therapeutic strategies, clinical outcomes remain inconsistent and appear to be influenced by dosing regimens, disease stage at intervention, and endpoint selection. Well-designed clinical studies are required to define their efficacy, optimal dosing, and disease-specific applicability.

## 1. Introduction

Neurodegenerative diseases (NDs) comprise a broad and heterogeneous group of neurological disorders characterized by progressive neuronal dysfunction and loss of specific neuronal populations in the central nervous system (CNS) or peripheral nervous system (PNS). These disorders also involve several interrelated cell populations and ultimately lead to cognitive impairment, motor dysfunction, a substantial reduction in quality of life, and premature death. Collectively, NDs pose a major public health challenge, and their global prevalence is projected to increase dramatically with population aging [1]. Major NDs include Alzheimer’s disease (AD), Parkinson’s disease (PD), amyotrophic lateral sclerosis (ALS), and Huntington’s disease (HD); multiple sclerosis (MS) is primarily an immune-mediated inflammatory demyelinating disease that nevertheless exhibits progressive neurodegeneration and is therefore frequently considered in discussions of neurodegenerative mechanisms [2]. Despite significant advances in understanding their underlying mechanisms, the multifactorial and convergent nature of neurodegenerative processes has hindered the development of effective disease-modifying therapies, leaving most current treatments primarily symptomatic rather than curative. Strategies aimed at mitochondrial function, oxidative stress control, and inflammation are of increasing interest [3].

A hallmark of neurodegenerative disorders is the convergence of several pathophysiological processes, including mitochondrial dysfunction, oxidative stress, calcium dysregulation, inflammation, protein misfolding, and the synaptic spread hypothesis [4]. Among these processes, oxidative stress and mitochondrial impairment appear to play particularly central roles in initiating and amplifying neuronal injury, whereas other mechanisms may exert more context-dependent effects [5]. Neurons, due to their high metabolic activity, limited regenerative capacity, and relatively reduced antioxidant defenses, are particularly vulnerable to disruptions in redox homeostasis and bioenergetic balance. Consequently, strategies targeting mitochondrial function and enhancing antioxidant defenses are of great interest, as alterations in these processes are associated with calcium dysregulation, neuroinflammation, protein misfolding, and neuronal death. Therefore, the modulation of these pathways is currently being intensively investigated as a promising therapeutic avenue [3,5].

In this context, nutraceuticals with multitarget mechanisms of action have attracted increasing attention. Nutraceuticals, a term derived from “nutrition” and “pharmaceutical”, are biologically active substances obtained from dietary sources that provide health benefits beyond basic nutrition. They play a role in disease prevention and treatment by influencing molecular pathways and physiological functions [6]. Two bioactive compounds, α-lipoic acid (ALA) and biotin (vitamin B7 or vitamin H), have gained increasing attention as promising neuroprotective agents, exhibiting complementary mechanisms that support cellular energy metabolism, attenuate oxidative stress, and modulate neuroinflammatory and signaling pathways. Both compounds are endogenously relevant cofactors involved in essential metabolic pathways; however, while pharmacological supplementation has demonstrated beneficial effects in preclinical models and selected clinical contexts, the strength and consistency of evidence across neurodegenerative diseases remain variable [7,8,9,10,11,12].

In this context, the central premise of the present review is that alpha-lipoic acid and biotin may provide complementary, multitarget support in neurodegenerative diseases by converging on partially overlapping mechanisms involved in mitochondrial function, redox homeostasis, inflammatory signaling, and neuroenergetic resilience. Rather than considering these compounds as isolated nutraceutical interventions, we examine whether their mechanistic intersection offers a biologically plausible framework for adjunctive or combination-oriented strategies across disorders such as Alzheimer’s disease, Parkinson’s disease, multiple sclerosis, amyotrophic lateral sclerosis, and Huntington’s disease. Accordingly, this review integrates mechanistic, preclinical, and clinical evidence to identify areas of overlap, translational limitations, and key gaps that should guide future research.

Importantly, while previous reviews have examined the individual roles of ALA or biotin in neurodegeneration, the present work differs by explicitly integrating their mechanistic intersection across convergent signaling pathways and by proposing a unified framework in which their complementary actions may enhance neuroenergetic resilience and inflammatory regulation. This integrative perspective aims to move beyond compound-specific descriptions toward a systems-level interpretation of multitarget metabolic interventions in neurodegenerative disease.

## 2. Materials and Methods

This manuscript presents a narrative and integrative review conducted in alignment with the Scale for the Assessment of Narrative Review Articles (SANRA), with the aim of ensuring transparency and methodological coherence in the synthesis of a heterogeneous body of evidence. Given the breadth and diversity of studies addressing ALA and biotin across neurodegenerative contexts, an integrative approach was considered appropriate to consolidate mechanistic insights and identify translational gaps. Accordingly, this review focuses on synthesizing mechanistic and translational evidence related to ALA and biotin in neurodegeneration, with particular emphasis on shared molecular pathways, potential complementary actions, and unresolved questions. Although the study was not designed as a systematic review or meta-analysis, a structured and reproducible search strategy was applied to reduce selection bias while preserving flexibility for mechanistic interpretation. Independent searches were performed in PubMed, Scopus, and Web of Science for English-language articles published between January 2000 and December 2025, using combinations of terms related to biotin, vitamin B7, α-lipoic acid, neurodegeneration, mitochondrial dysfunction, oxidative stress, neuroinflammation, and relevant signaling pathways (e.g., cGMP, AMPK, NRF2, NF-κB, Akt). This strategy yielded several hundred records, from which a focused subset of approximately 60–70 primary and clinically relevant studies was retained for qualitative integrative analysis following title and abstract screening, duplicate removal, and full-text assessment. Eligible sources included peer-reviewed mechanistic studies, preclinical experimental models (in vitro, in vivo, and ex vivo), and clinical investigations, both randomized and non-randomized, as well as selected conceptual articles providing mechanistic context. Study selection relied on expert judgment and was constrained by language, time frame, and heterogeneity of models and outcomes. To enhance interpretability, the evidence was organized by experimental model and disease category, facilitating cross-model comparisons, identification of convergent mechanisms, and critical appraisal of current limitations and knowledge gaps.

## 3. Results

### 3.1. Alpha-Lipoic Acid

Alpha (α)-lipoic acid (ALA) [thioctic acid; dithioctanoic acid; IUPAC: 5-(1,2-dithiolan-3-yl)pentanoic acid] is an organosulfur compound with a single chiral center at C6, generating R- and S-enantiomers; only the R-form is synthesized endogenously in mammalian mitochondria via lipoic acid synthase from octanoic acid and cysteine, although in insufficient quantities to sustain metabolic requirements [11]. Consequently, exogenous intake becomes necessary despite ALA not being classified as an essential nutrient. No formal dietary requirement exists, yet an approximate intake of 30 mg/day is often referenced in the literature [13]. Biochemically, ALA functions as a covalently bound cofactor of mitochondrial enzyme complexes, including pyruvate dehydrogenase (PDH), α-ketoglutarate dehydrogenase (KGDH), branched-chain α-keto acid dehydrogenase (BCKDH), and the 2-oxoadipate dehydrogenase complex (OADH/DHTKD1), which play a central role in oxidative decarboxylation and cellular energy metabolism. Through these actions, ALA supports TCA cycle flux, acetyl-CoA availability, and neuronal energy homeostasis, processes that are frequently compromised during aging and in neurodegenerative diseases [12].

Dietary ALA is absorbed via sodium-dependent multivitamin transporter (SMVT) and monocarboxylate transporters (MCTs) [11,12]. The presence of these transporters in human brain microvessels supports ALA’s capacity to cross the blood–brain barrier (BBB) and reach neural tissue [14]. Its amphipathic nature enables distribution in both aqueous and lipid environments and facilitates penetration across the BBB, allowing direct effects on neurons and glia [11]. At pharmacological doses (≈600–2400 mg/day), plasma concentrations are sufficient to exert systemic and central nervous system (CNS) effects, making it a suitable candidate for treating neurodegenerative disorders characterized by mitochondrial dysfunction and oxidative stress [12,15].

ALA and its reduced form, dihydrolipoic acid (DHLA), form a redox couple capable of directly scavenging reactive oxygen, nitrogen, and sulfur species (ROS/RNS/RSS), regenerating endogenous antioxidants such as glutathione and vitamins C and E, and chelating redox-active metals (Fe, Cu, Zn, Hg, Pb) [10,11,12]. These mechanisms restore redox homeostasis, a central deficit in Alzheimer’s, Parkinson’s, and other neurodegenerative diseases [1,3,5]. In addition, ALA activates the NRF2/ARE pathway, inducing the expression of cytoprotective enzymes like heme oxygenase-1 (HO-1) and NAD(P)H:quinone oxidoreductase 1 (NQO1), while suppressing NF-κB-mediated transcription of pro-oxidant cytokines [12]. Although some evidence derives from non-neural systems (e.g., IL-8 suppression in *Helicobacter pylori*-infected epithelial cells), these findings reinforce ALA’s systemic antioxidant and anti-inflammatory potential relevant to CNS disorders [16].

Beyond its redox and metabolic actions, an epigenetically relevant aspect of α-lipoic acid activity has been proposed. Rather than involving covalent histone modification, experimental evidence indicates that reduced lipoic acid can inhibit histone deacetylases, leading to increased acetylation of HDAC substrates and modulation of gene expression. Although the physiological relevance and tissue specificity of these effects, particularly in neural contexts, remain to be fully clarified, this mechanism suggests that part of ALA’s immunometabolic actions may extend to chromatin-associated regulatory processes [17].

In BV-2 microglia, ALA has been shown to inhibit MAPK pathways (p38, JNK, ERK) and prevent NLRP3 inflammasome assembly, thereby reducing IL-1β and IL-18 secretion and limiting oxidative–nitrosative stress amplification [18]. In subarachnoid hemorrhage models, ALA has been reported to attenuate STING–TBK1–NF-κB signaling and associated inflammatory responses [19]. Furthermore, ALA elevates intracellular cAMP and activates PKA, providing an additional anti-inflammatory regulatory mechanism in glial and leukocyte responses [20]. In a dapsone-induced neurotoxicity model, ALA was reported to reduce markers associated with hippocampal microgliosis and astrogliosis, restore BDNF levels, and reduce oxidative injury, thereby highlighting its neuroimmune modulatory role [21].

ALA improves mitochondrial function by enhancing Akt phosphorylation and suppressing JNK activity, leading to PGC-1α upregulation and induction of oxidative phosphorylation genes, mitochondrial biogenesis, and ATP restoration in aging brain tissue [22]. This effect depends on SIRT1, as its inhibition abolishes ALA-induced PGC-1α activation, implicating the SIRT1–PGC-1α axis in neuroprotection [23]. ALA has also been shown to enhance mitophagy and shift APP processing toward the non-amyloidogenic pathway, reducing amyloid-β accumulation and improving cognitive function in APP23/PS45 transgenic mice [24]. ALA, in combination with vitamin D_3_, also reduced astrocytic oxidative stress and iron accumulation, thereby preserving astrocyte viability and supporting brain aging resistance in vitro [25]. In Schwann cells exposed to high glucose, ALA increases the Bcl-2/Bax ratio and suppresses caspase-3 activation, supporting its mitochondrial and anti-apoptotic role [26].

It should be noted that several cellular markers commonly used to characterize glial activation are not entirely cell-type specific, and their interpretation should therefore be considered within the broader experimental context of each study. Importantly, microglial activation does not represent a binary state but rather a dynamic spectrum of functional phenotypes shaped by local microenvironmental signals. Likewise, astrocytes exhibit marked regional and functional heterogeneity, with context-dependent neuroprotective or neurotoxic roles. Accordingly, changes in marker expression should be interpreted with caution and should not be assumed to reflect a single, uniform cellular activation state.

ALA’s signaling actions integrate survival and metabolism. In dopaminergic stress models, ALA activates PI3K–Akt, improves motor behavior, reduces neuroinflammation, and diminishes caspase-3 activation, identifying Akt as a proximal survival mediator under mitochondrial toxin load [27]. Consistent with its cytoprotective profile, ALA ameliorates aging-related impairments in cerebral energy metabolism by enhancing Akt activation, attenuating JNK signaling, and robustly upregulating PGC-1α, thereby supporting bioenergetic homeostasis and neuronal resilience [28]. This notion aligns with clinical and preclinical evidence summarized by Capece et al. [29], who highlight that ALA improves glucose utilization, enhances mitochondrial efficiency, and strengthens redox-sensitive signaling networks that collectively support cellular bioenergetics and metabolic resilience [29]. In models of diabetic peripheral neuropathy, ALA exerts cytoprotective actions primarily through AMPK activation, leading to attenuation of oxidative stress and suppression of apoptosis. By restoring antioxidant capacity and mitochondrial redox balance, ALA contributes to improved neuronal integrity independently of classical insulin-signaling pathways [30]. Importantly, in diabetic peripheral neuropathy, ALA also activates AMPK to counter oxidative stress and caspase-mediated apoptosis; although this effect is observed outside the CNS, it is mechanistically relevant to neurodegenerative disorders where AMPK dysfunction contributes to neuronal vulnerability [31].

Taken together, available evidence indicates that ALA exerts neuroprotective effects through integrated modulation of redox balance, inflammatory signaling, mitochondrial bioenergetics, and insulin/Akt/AMPK pathways. Its ability to simultaneously activate NRF2, inhibit NF-κB and NLRP3, restore mitochondrial function via PGC-1α and SIRT1, and improve neuronal glucose metabolism via Akt and AMPK positions ALA as a pleiotropic therapeutic candidate in neurodegenerative disorders.

Overall, these findings position ALA as a pleiotropic modulator of redox balance, mitochondrial efficiency, inflammatory signaling, and cell-survival pathways. This mechanistic profile provides a useful reference point for examining whether biotin, despite acting through distinct biochemical routes, converges on complementary neuroprotective processes relevant to neurodegeneration.

### 3.2. Biotin

Biotin (vitamin B7 or vitamin H) is a water-soluble member of the B-complex family. Structurally, it is a heterocyclic compound consisting of a 2-oxohexahydro-1H-thieno [3,4-d]imidazole core linked to a valeric acid side chain through a tetrahydrothiophene ring. From a biological standpoint, biotin is an essential cofactor for several ATP-dependent carboxylases, including acetyl-CoA carboxylases 1 and 2 (ACC1 and ACC2), pyruvate carboxylase (PC), propionyl-CoA carboxylase (PCC), and 3-methylcrotonyl-CoA carboxylase (MCC), thereby supporting central metabolic processes such as gluconeogenesis, fatty acid synthesis, and branched-chain amino acid catabolism [7,9]. It is obtained primarily from dietary sources and the intestinal microbiota and is absorbed in the small intestine via the sodium-dependent multivitamin transporter (SMVT). The same transporter also facilitates biotin uptake into the central nervous system by mediating its transport across the blood–brain barrier [7,8,9]. Through these roles, it contributes to metabolic homeostasis, particularly in tissues with high energetic demand such as the central nervous system [32]. Impaired biotin transport, as observed in biotinidase or holocarboxylase synthetase deficiencies, results in neurological manifestations such as seizures, developmental delay, and hypotonia, underscoring its essential role in brain function [33].

Biotin plays a crucial role in neurobiological functions within the central nervous system through distinct biochemical and regulatory mechanisms [32]. Its carboxylase-dependent activity supports tricarboxylic acid (TCA) cycle anaplerosis through PC and regulates lipid metabolism via ACC1 and ACC2, processes that influence neuronal membrane composition, myelin synthesis, and cellular energy balance [9,32,34]. Disruption of these pathways has been linked to impaired neurotransmission and demyelination, providing a mechanistic rationale for exploring high-dose biotin as a supportive intervention in demyelinating disorders, although this rationale should not be interpreted as definitive clinical proof [32,34,35].

At pharmacological doses (2–300 mg/kg), which markedly exceed the daily requirement of 5–40 μg, biotin has been shown to exert broader regulatory effects on cellular physiology. These effects include modulation of carbohydrate and lipid metabolism, immune-related pathways, and cellular differentiation, and are largely mediated by coordinated changes in gene expression [7,9]. Transcriptomic analyses indicate that biotin status can influence the expression of more than 2000 genes in humans and experimental models, including genes involved in metabolism, oxidative stress responses, and inflammatory signaling [7,9,36]. In addition, an epigenetic component has been proposed, as biotin can be covalently attached to specific lysine residues of histones H2A, H3, and H4 via the action of holocarboxylase synthetase and biotinidase [36,37]. Although the physiological relevance of histone biotinylation remains debated, in part due to its relatively low abundance compared with other histone modifications [7,9], these findings highlight a potential epigenetic dimension to biotin’s neuroprotective actions, particularly in demyelinating and neurodegenerative disorders [9,32,34]. Moreover, recent evidence from a murine model of malnutrition suggests that biotin may also shape fetal metabolic programming through epigenetic regulation [38]. Together, these observations suggest that pharmacological biotin may influence cellular function through regulatory layers that extend beyond classical intermediary metabolism.

In addition to these metabolic and epigenetic effects, a central signaling pathway implicated in the pharmacological actions of biotin is the nitric oxide (NO)–soluble guanylate cyclase (sGC)–cyclic guanosine monophosphate (cGMP) axis. Early biochemical studies demonstrated that biotin and biotin analogs stimulate sGC activity in several rat tissues, including the cerebellum, as well as in human lymphoid cells, where biotin availability increases NO production, sGC activity, intracellular cGMP levels, and downstream protein kinase G (PKG) signaling [7,9]. Functional evidence supporting this pathway has been obtained in pancreatic islets, where biotin-induced gene expression is abolished by sGC or PKG inhibition and reproduced by cGMP analogs, indicating a causal role for cGMP signaling and downstream engagement of PI3K–Akt pathways through autocrine insulin release [39]. In vascular models, biotin enhances vasorelaxation and attenuates Ca^2+^-dependent contraction in rat aortic rings, consistent with modulation of Ca^2+^ influx and intracellular Ca^2+^ handling [40]. Although demonstrated in peripheral tissue, this Ca^2+^-sparing effect aligns with the proposed modulation of NO–sGC–cGMP signaling and may extend to the neurovascular unit. Given that disturbed Ca^2+^ homeostasis is a central pathogenic denominator in neurodegenerative diseases, driving mitochondrial depolarization, excitotoxic injury, and impaired synaptic plasticity, biotin’s capacity to stabilize Ca^2+^ dynamics could confer additional neuroprotective leverage.

In neural contexts, cyclic guanosine monophosphate (cGMP)-dependent signaling is closely linked to neuronal survival and synaptic plasticity. Elevation of this second messenger engages PKG-dependent pathways, including PI3K–Akt/mTOR signaling, and facilitates long-term potentiation (LTP) when intracellular levels rise postsynaptically in hippocampal neurons [41,42]. Disruption of this signaling network has been implicated in Alzheimer’s disease, where oligomeric amyloid-β suppresses the physiological increase associated with LTP induction and impairs CREB phosphorylation [43]. Pharmacological strategies that restore this pathway—such as NO donors, sGC stimulation, cGMP analogs, or phosphodiesterase-5 inhibition—have been shown to rescue synaptic plasticity and memory performance in experimental models [43,44]. Accordingly, if biotin enhances sGC-dependent signaling in neural tissue, it could plausibly engage the same PKG–CREB network and intersect with PI3K–Akt/ERK pathways that support synaptic resilience, particularly given the established contribution of PI3K dysregulation to Alzheimer’s disease pathology.

Beyond synaptic signaling, biotin has also been linked to cellular bioenergetics and mitochondrial support, particularly in glial cells. In this context, cyclic nucleotide-dependent signaling and metabolic pathways have been implicated in oligodendrocyte function and myelin-related processes, supporting the notion that intracellular signaling networks contribute to oligodendrocyte lineage behavior and resilience [34,35]. Complementary in vitro evidence from Sghaier et al. [45] demonstrated in 158N murine oligodendrocytes that biotin attenuates oxidative stress, preserves mitochondrial integrity, and prevents 7β-OHC-induced oxiapoptophagy [45]. Although this study did not assess cGMP directly, it reinforces the concept of a cytoprotective environment that enables oligodendrocytes to respond more effectively to reparative cues, including cGMP-mediated pathways [7,9,45] which is a mechanism of particular relevance in progressive forms of MS [34,35].

In peripheral metabolic models, biotin-induced increases in cGMP have been associated with activation of AMP-activated protein kinase (AMPK), a master regulator of cellular energy homeostasis that inhibits key lipogenic enzymes, thereby reducing lipid accumulation, and indirectly supports mitochondrial function. Although demonstrated mainly in hepatic and adipocyte systems, this AMPK-mediated mechanism is mechanistically relevant to neuroenergetics, given the central role of mitochondrial dysfunction and energy failure in neurodegenerative disorders [46,47]. Consistent with this rationale, high-dose biotin improves mitochondrial function, oxidative phosphorylation efficiency, and redox balance in experimental models of adrenoleukodystrophy and tauopathy, and exerts similar protective effects in oligodendrocytes [45,48,49,50]. Moreover, biotin has been evaluated as part of multi-nutrient strategies that enhance mitochondrial enzyme activity and metabolic homeostasis in peripheral disease models, supporting its broader relevance within bioenergetic interventions [51].

Additionally, biotin supplementation has been shown to modulate inflammatory and immune processes, with evidence indicating context-dependent immunoregulatory effects. Early human studies demonstrated that biotin influences immune cell activity and cytokine profiles: Zempleni et al. reported reduced proliferation of peripheral blood mononuclear cells and decreased secretion of pro-inflammatory cytokines such as IL-1β and IL-2 following biotin supplementation, whereas Wiedmann et al. observed increased IFN-γ and IL-1β mRNA levels accompanied by reduced IL-4 expression, suggesting a shift toward a Th1-oriented immune response [52,53]. Together, these findings indicate that biotin can fine-tune cytokine expression and immune polarization rather than acting as a uniformly immunosuppressive agent.

Complementary experimental evidence supports a broader role for biotin in limiting inflammatory signaling. In a propionic acid-induced rat model of autism, Sahin et al. showed that magnesium biotinate markedly attenuated oxidative stress and neuroinflammation, restoring antioxidant enzyme activities, reducing lipid peroxidation, and significantly downregulating pro-inflammatory cytokines including TNF-α, IL-6, and IL-17 in brain tissue, with concomitant improvement in neuronal integrity and behavioral outcomes [54]. In parallel, studies in peripheral inflammatory disease models have demonstrated that biotin supplementation suppresses NF-κB activation and reduces downstream pro-inflammatory cytokine production, particularly in intestinal inflammation, whereas biotin deficiency exacerbates NF-κB-dependent inflammatory responses [55,56]. In a murine model of dextran sulfate sodium-induced colitis, biotin supplementation was shown to attenuate NF-κB activation, reduce pro-inflammatory cytokine expression, and improve intestinal barrier integrity, providing direct experimental evidence that biotin can modulate inflammatory transcriptional programs in vivo [57]. Narrative and mechanistic reviews further support the concept that biotin status influences immune and inflammatory transcriptional programs through regulation of key signaling pathways rather than through direct antioxidant activity [58].

Although direct evidence for biotin-mediated regulation of vascular adhesion molecules such as ICAM-1 or VCAM-1 in cerebral microvessels is currently lacking, the available data nonetheless position biotin as a modulator of neurovascular inflammation. By attenuating NF-κB-dependent inflammatory signaling and limiting pro-inflammatory cytokine output, biotin may indirectly restrain endothelial activation and leukocyte recruitment within the cerebral microenvironment [59]. Consistent with this view, Moretti and Peinkhofer emphasized the importance of B-vitamin status, including biotin, for maintaining endothelial integrity and microvascular resilience, particularly in the context of small-vessel dysfunction [60]. Moreover, biotin-induced elevations in cGMP, with subsequent engagement of PKG-dependent survival pathways, may intersect with these vascular and inflammatory effects by dampening microglial activation, enhancing neuronal resistance to oxidative and inflammatory stress, and counteracting molecular cascades implicated in neurodegeneration.

Finally, evidence from immune cell models underscores the relevance of adequate biotin availability for immune homeostasis. Although these findings derive from conditions of biotin deficiency rather than supplementation, Agrawal et al. demonstrated that biotin deprivation in human dendritic cells triggers a marked overproduction of pro-inflammatory cytokines, including IL-1β, TNF-α, IL-12p40, and IL-23, through NF-κB activation, thereby promoting a Th1/Th17-skewed immune profile [61]. Collectively, these findings support the view that biotin participates in the regulation of peripheral and central inflammatory signaling, providing a mechanistic framework through which biotin-dependent immunomodulation may indirectly influence neuroinflammatory processes relevant to central nervous system pathology.

These observations support the hypothesis that biotin may lessen neuronal vulnerability to oxidative injury and metabolic stress. The available evidence indicates that biotin exerts pleiotropic effects through multiple converging mechanisms, including enhancement of cellular energy metabolism, epigenetic regulation of gene expression, attenuation of oxidative stress, and modulation of intracellular signaling and inflammatory pathways. Taken together, these mechanisms are biologically relevant to processes underlying neuronal vulnerability and degeneration and provide a conceptual framework for understanding the potential neuroprotective actions of biotin, which will be further discussed in a translational and clinical context below.

Thus, although biotin differs from ALA in its primary biochemical functions, the available evidence suggests that both compounds intersect at several pathogenic nodes relevant to neurodegeneration, particularly mitochondrial support, metabolic regulation, inflammatory modulation, and cellular stress resilience. This conjunction provides the rationale for the following section, which examines their probable complementary and potentially additive interactions.

### 3.3. Probable Complementary and Potentially Additive Interaction Between Alpha-Lipoic Acid and Biotin: Molecular Rationale for Neuroprotection in Neurodegenerative Diseases

**Figure 1:**
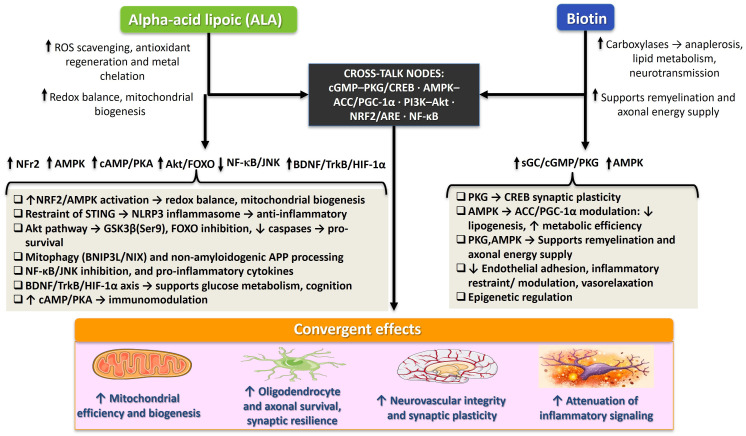
Alpha-Lipoic acid (**left**) and biotin (**right**) are shown to engage partially overlapping signaling nodes, including cGMP–PKG/CREB, AMPK–ACC/PGC-1α, PI3K–Akt, NRF2/ARE, and NF-κB, which collectively coordinate redox homeostasis, mitochondrial efficiency and biogenesis, metabolic reprogramming, and attenuation of inflammatory signaling. Downstream convergence of these pathways may support oligodendrocyte and axonal survival, neurovascular integrity, and synaptic plasticity, thereby contributing to an integrated neuroprotective effect. The figure was created using “Smart Servier Medical Art” and “PowerPoint”.

A biologically plausible complementary and potentially additive interaction between ALA and biotin in neurodegenerative disease is suggested by their actions on distinct yet intersecting molecular nodes that govern neuronal resilience under oxidative, inflammatory, and bioenergetic stress. As summarized in Figure 1, alpha-lipoic acid and biotin converge on partially overlapping signaling networks—including cGMP–PKG/CREB, AMPK–ACC/PGC-1α, PI3K–Akt, NRF2/ARE, and NF-κB—that together shape redox homeostasis, mitochondrial efficiency and mitochondrial biogenesis, and inflammatory restraint. This downstream convergence may contribute to oligodendrocyte and axonal resilience, neurovascular integrity, and synaptic function under degenerative stress. Importantly, it provides a biologically grounded framework for future combination studies and for the selection of pharmacodynamic readouts, such as cerebrospinal fluid or plasma cGMP levels and the induction of NRF2-responsive genes.

At the level of mitochondrial metabolism, ALA directly supports oxidative decarboxylation by serving (as lipoamide) in PDH, α-KGDH, and BCKDH complexes, stabilizing flux into the TCA cycle and acetyl-CoA production essential for neuronal ATP generation [12]. Biotin complements this by sustaining anaplerosis via PC and regulating malonyl-CoA through ACC1/ACC2, thereby coordinating fatty-acid synthesis and oxidation, membrane lipid renewal, and substrate availability for myelin and synapses [7,8,9,32]. In disease-relevant models, pharmacological biotin restores oxidative phosphorylation, ΔΨm, and ATP while limiting ROS accumulation in neurons and axons and oligodendroglia [45,48,49]. In parallel, ALA promotes PGC-1α-driven transcriptional programs and mitochondrial biogenesis through Akt-JNK balance and SIRT1 dependence, improving electron-transport efficiency and ATP output in aging brain tissue [22,23,25]. These complementary inputs—substrate management and anaplerosis (biotin) together with dehydrogenase function and biogenic reprogramming (ALA)—support the hypothesis of additive, rather than strictly synergistic, restoration of mitochondrial throughput under degenerative stress. Consistent with this view, biotin enhances vasorelaxation and reduces Ca^2+^-dependent contraction in rat aortic rings, suggesting attenuation of Ca^2+^ influx and release [40]. Such Ca^2+^-stabilizing effects could complement α-lipoic acid’s antioxidant and AMPK-activating properties, jointly counteracting Ca^2+^ dysregulation and mitochondrial stress.

In redox biology, the ALA/DHLA couple directly quenches ROS/RNS/RSS, regenerates glutathione and vitamins C/E, and chelates redox-active metals, while also activating NRF2/ARE to induce HO-1 and NQO1 and restrain pro-oxidant cytokine programs [10,11,12]. Although biotin is not a canonical thiol redox buffer, it reduces ROS and preserves ΔΨm in oligodendrocytes and neurodegeneration-relevant models, and can remodel gene expression/epigenetic states that favor stress tolerance [36,37,38,45]. Consistent with this antioxidant profile, biotin supplementation in high-fructose-fed rats significantly attenuated hepatic lipid peroxidation [62]. Although observed in liver tissue rather than neural cells, it mechanistically supports biotin’s role in restraining oxidative damage via improved mitochondrial redox handling and reduced peroxidative stress and is mechanistically relevant to neuroenergetic contexts. The working expectation is that ALA supplies immediate chemical and NRF2-linked antioxidant capacity, while biotin stabilizes mitochondrial–metabolic set points that may lower oxidant production at source, constituting a two-lever approach to redox homeostasis.

For neuroinflammation, ALA suppresses NF-κB and MAPK (p38/JNK/ERK), reduces NLRP3 inflammasome activity, and, in vascular–immune interfaces, can impose a cAMP/PKA “brake” on leukocyte–glia activation [18,19,20,21,63]. Biotin modulates cytokine programs and has been associated with reduced endothelial adhesion molecule expression and leukocyte trafficking in the cerebral microvasculature, an anti-inflammatory profile relevant to MS and small-vessel pathology. Notably, these effects are not explicitly mediated by cGMP in those reports [59,60]. Importantly, biotin deficiency biases NF-κB-dependent cytokine production, as demonstrated in human dendritic cells where low biotin levels enhance IL-1β, TNF-α, IL-12p40 and IL-23 expression through NF-κB activation (non-CNS evidence that supports a generalizable mechanism) [61]. Additional evidence from non-neural systems further supports biotin’s immunomodulatory capacity: pharmacologic supplementation decreases PBMC proliferation and reduces pro-inflammatory cytokine release [52], while also shifting cytokine transcription toward a Th1 profile with increased IFN-γ and IL-1β and reduced IL-4 expression [53]. These systemic effects align with the broader anti-inflammatory framework attributed to biotin. Within this framework, one prevailing hypothesis for high-dose biotin in progressive MS proposes that its benefits arise from anti-inflammatory and pro-myelinating effects mediated by cGMP signaling in the neurovascular unit and glia [32,34,59]. Together, these actions suggest layered control of inflammatory checkpoints: ALA acting on canonical inflammatory hubs and inflammasome assembly; biotin damping vascular-adhesive and cytokine milieus that feed neuroimmune activation.

Convergence is especially apparent in neuroenergetic signaling. ALA engages PI3K–Akt to support survival signaling and damp apoptotic execution, and (in metabolic contexts) improves insulin-pathway tone while limiting redox-sensitive phosphatases (PTEN/PP2A/PTP1B) [27,28,30]. Biotin couples to sGC–cGMP in peripheral and cellular systems and can crosstalk with PI3K–Akt/ERK and CREB pathways that underwrite synaptic plasticity [39,41,42,43,44]. Crucially, biotin-induced cGMP elevations activate AMPK, and AMPK activity suppresses ACC1/FAS, rebalances lipid flux, and supports mitochondrial quality control, mechanisms relevant to neuroenergetics even when shown outside the CNS [46,47]. ALA also interfaces with the AMPK–SIRT1–PGC-1α axis (directly or via SIRT1-dependent PGC-1α induction) to expand oxidative capacity [22,23]. Thus, both agents converge on AMPK–SIRT1–PGC-1α and PI3K–Akt nodes, suggesting coordinated enhancement of bioenergetic resilience, anti-apoptotic bias (↑Bcl-2/Bax, ↓caspase-3), and an activity-dependent plasticity program.

A further point of intersection lies in myelin biology and the neurovascular unit. Biotin’s ACC-linked lipid synthesis and oligodendroglial support align with remyelination-oriented mechanisms in progressive MS [34], while ALA’s mitochondrial and anti-inflammatory effects in glia and endothelium may preserve the bioenergetic and immune environment needed for effective myelin maintenance [18,20,21,24,63]. Consistent with earlier observations, biotin enhances vasorelaxation and reduces Ca^2+^-dependent contraction in peripheral vessels (peripheral evidence), consistent with improved microvascular tone relevant to oxygen-glucose delivery in compromised tissue [40,59,60]. Together, these actions protect the axon–myelin–endothelium triad that degenerates across disorders.

At the apoptotic/survival axis, both molecules converge on preservation of neuronal viability. ALA increases the Bcl-2/Bax ratio, reduces cytochrome c release, and suppresses cleaved caspase-3 in neuronal and glial models exposed to oxidative or metabolic stress [26,64,65]. In rotenone-induced Parkinsonian models, ALA-driven PI3K–Akt activation inhibits pro-apoptotic GSK3β and restrains caspase-9/BAD signaling, reducing dopaminergic neuron loss [27,28]. Although biotin is less extensively studied in apoptosis regulation, oligodendroglial models show that it prevents 7β-hydroxycholesterol-induced oxiapoptophagy and preserves mitochondrial membrane potential [45]. These findings support coordinated anti-apoptotic effects where ALA directly activates survival kinases and inhibits mitochondrial depolarization, while biotin preserves metabolic stability to prevent the upstream triggers of apoptosis.

Although central nervous system data on the combination are lacking, peripheral neuropathy studies suggest efficacy of nutrient-based interventions: ALA plus methylcobalamin improved neuropathic pain and nerve conduction [66], and a fixed formulation containing ALA, biotin, and B-vitamins demonstrated clinical benefit with good tolerability [67]. A rational combination strategy would pair ALA (for immediate redox buffering, NRF2 induction, NF-κB/NLRP3 restraint, and PI3K–Akt/AMPK–SIRT1–PGC-1α engagement) with biotin (for anaplerosis, ACC-guided lipid metabolism/remyelination support, sGC–cGMP–AMPK coupling, and epigenetic/transcriptional tuning). Importantly, direct experimental evidence demonstrating truly complementary or additive pharmacological effects between ALA and biotin remains limited. Well-designed combinatorial approaches, supported by quantitative interaction analyses (e.g., additivity or synergy models), will be required to determine whether their combined effects exceed those predicted from independent actions.

Such approaches should incorporate factorial preclinical designs and biomarker-driven strategies to distinguish additive, synergistic, or antagonistic responses. In this context, the integration of pharmacodynamic biomarkers—such as cGMP signaling activity, NRF2-responsive gene expression, and mitochondrial bioenergetic parameters—will be essential to establish whether combined ALA–biotin interventions provide measurable advantages over single-compound approaches. Altogether, these strategies may help bridge the gap between mechanistic plausibility and translational validation.

Having established the main mechanistic points of overlap between ALA and biotin, the next section examines whether these theoretical intersections are reflected in preclinical disease models, where oxidative, inflammatory, and mitochondrial disturbances can be assessed in integrated biological contexts.

### 3.4. Preclinical Evidence on Alpha-Lipoic Acid and Biotin in Neurodegenerative Disorders

Preclinical studies using in vitro systems and animal models provide convergent evidence supporting the neuroprotective effects of ALA and biotin across several major neurodegenerative diseases. The key findings are summarized below by disorder, with emphasis on the experimental context and the underlying mechanistic insights.

#### 3.4.1. Alzheimer’s Disease

Oxidative stress and mitochondrial dysfunction are central contributors to Alzheimer’s disease (AD) pathology, together with β-amyloid (Aβ) aggregation and tau hyperphosphorylation [3,4]. Beyond these classical mechanisms, accumulating preclinical evidence highlights the pleiotropic neuroprotective actions of ALA. Across multiple experimental models of AD, these effects have been associated with attenuation of cognitive decline and improvement of memory-related outcomes. Such neuroprotective actions are mediated through multiple mechanisms, such as the attenuation of oxidative stress, preservation of mitochondrial function, modulation of neuroinflammatory pathways, and a reduction in amyloid-β-associated toxicity [11,12,15,68,69]. In neuronal cultures overexpressing APP, ALA enhanced mitochondrial bioenergetics by increasing the activity of respiratory complexes, stabilizing mitochondrial membrane potential, and sustaining ATP production [70]. Consistent with these findings, transgenic AD models have demonstrated multi-level neuroprotective actions. In Tg2576 mice, chronic dietary supplementation attenuated age-related impairments in hippocampal-dependent memory and restored deficits in long-term potentiation, indicating a partial rescue of synaptic plasticity mechanisms disrupted in amyloidogenic contexts [71]. Independently, in a triple-transgenic AD model, ^13C-NMR metabolic flux analyses revealed a reversal of pronounced metabolic impairments, including deficits in mitochondrial oxidative metabolism and substrate utilization, further supporting the capacity of ALA treatment to normalize bioenergetic dysfunction central to AD pathophysiology [72]. More recent studies have expanded these mechanistic insights. In APP23/PS45 mice, treatment enhanced mitophagy and promoted non-amyloidogenic APP processing, thereby strengthening synaptic resilience and neuronal survival [24]. In tauopathy P301S mice, chronic administration restored cerebral glucose metabolism through activation of the BDNF/TrkB/HIF-1α axis, leading to measurable cognitive improvement [73]. Moreover, in BV-2 microglia, ALA increased phagocytosis of oligomeric Aβ1–42 via a COX-2/PPARγ/CD36 pathway, underscoring its immunomodulatory potential [74]. Overall, these findings support the biological plausibility of ALA as a multitarget mitochondrial and redox modulator in Alzheimer’s disease. However, whether these effects translate into disease-modifying clinical benefit remains unresolved and will require confirmation in adequately powered randomized controlled trials.

In contrast, preclinical studies exploring biotin in AD are limited but suggest potential benefits via mitochondrial and metabolic support. In a tauopathy model, Lohr et al. [49] demonstrated that tau expression disrupts biotin homeostasis and mitochondrial function, while biotin supplementation rescued mitochondrial deficits and improved neuronal viability, with reduced biotinylated carboxylases also observed in AD human brain tissue. In an amyloid-β (Aβ)-induced rat model, oral biotin given before and after Aβ injection improved memory performance, reduced oxidative stress, and decreased hippocampal plaque load [75]. Overall, available data suggest that this compound may protect against AD-related pathology primarily by restoring mitochondrial function and promoting bioenergetic resilience, although direct evidence in transgenic AD models remains scarce.

#### 3.4.2. Parkinson’s Disease

In Parkinson’s disease (PD), degeneration of dopaminergic neurons in the substantia nigra is driven by oxidative stress, mitochondrial dysfunction, and α-synuclein aggregation [2,4]. In toxin-induced models of Parkinson’s disease, α-lipoic acid (ALA) exhibits marked neuroprotective properties. In 6-hydroxydopamine (6-OHDA)-lesioned rats, treatment attenuated dopaminergic neuron loss, reduced oxidative stress markers, and improved motor coordination, supporting an antioxidant and restorative profile [76]. Likewise, in MPTP/MPP^+^-induced models and PC12 neuronal cells, ALA enhanced SIRT1 and PGC-1α expression, mitigated oxidative injury, and preserved dopaminergic integrity, highlighting the involvement of the SIRT1–PGC-1α signaling axis in neuroprotection [77]. In 6-OHDA-induced models of Parkinson’s disease, modulation of iron metabolism and limitation of iron accumulation have also been reported, mechanisms increasingly linked to ferroptotic vulnerability [78]. More recently, these findings have been extended to the attenuation of motor deficits through inhibition of ferroptosis, a process now recognized as a relevant contributor to Parkinson’s disease pathogenesis [79]. In a unilateral 6-hydroxydopamine (6-OHDA) rat model of Parkinson’s disease, de Araújo et al. [80] found that oral administration of ALA (100 mg/kg or 200 mg/kg) significantly improved motor behavior (reduced apomorphine-induced rotations, increased locomotion and contralateral paw use) and attenuated markers of oxidative stress (lipid peroxidation, nitrite levels, catalase activity) [80].

Beyond disease-modifying effects, ALA reduces L-DOPA-induced dyskinesia in 6-OHDA rats through attenuation of oxidative stress without diminishing antiparkinsonian efficacy, suggesting potential as an adjunct symptomatic strategy [81]. Recent formulation approaches have expanded the neuroprotective potential of ALA by improving its pharmacological stability and brain bioavailability. Notably, novel nanomicellar complexes combining carnosine and ALA have demonstrated significant efficacy in rotenone-induced models of Parkinson’s disease, where they enhanced antioxidant activity, reduced neuroinflammation, and ameliorated behavioral impairments, thereby underscoring the advantages of nanoscale delivery systems for targeting dopaminergic degeneration [82]. In parallel, studies in rotenone-exposed mice revealed that ALA activates the PI3K/Akt signaling cascade, counteracts oxidative stress, and inhibits caspase-3-mediated apoptosis, resulting in improved motor coordination and neuronal survival [27]. Taken together, these findings position ALA as a multi-target mitochondrial and iron-modulating agent with reproducible benefits across rodent and cellular PD models, but they also warrant more rigorous evaluation of dosing, pharmacokinetics, and head-to-head comparisons in modern genetic and toxin paradigms before progression toward early-phase clinical testing.

Biotin’s potential in PD remains largely unexplored; however, its established roles in mitochondrial bioenergetics and fatty acid metabolism suggest possible indirect support for dopaminergic survival. The strongest evidence derives from manganese (Mn)-induced parkinsonism models in *Drosophila* and human iPSC-derived midbrain dopaminergic neurons, where dietary biotin supplementation attenuated Mn-induced motor deficits, neuronal loss, and mitochondrial dysfunction, while biotinidase knockdown exacerbated toxicity [83]. By contrast, no rodent studies to date have directly evaluated biotin in canonical toxin- or genetic-based PD models such as MPTP, 6-OHDA, rotenone, or α-synuclein overexpression. This gap is notable given the broad availability of such models for mechanistic and therapeutic testing [84]. Overall, evidence for biotin in PD is still preliminary and limited to selected systems; confirmation in standard rodent models will be essential before advancing therapeutic claims.

#### 3.4.3. Multiple Sclerosis

Multiple sclerosis (MS) is a chronic immune-mediated disease of the central nervous system, characterized by immune-driven myelin loss, inflammation, and neuronal damage that disrupt neural signaling [2,4]. In experimental autoimmune encephalomyelitis (EAE) models, preclinical studies have shown that ALA exerts neuroprotective and anti-inflammatory effects by reducing clinical severity scores, alleviating nociceptive behaviors, stabilizing blood–brain barrier integrity, and downregulating inflammatory mediators [23]. Jones et al. [85] reported that treatment with ALA, alone or in combination with associative conditioning, significantly reduced disease severity and leukocyte infiltration into the spinal cord, underscoring its immunomodulatory and neuroprotective potential in autoimmune demyelination [85]. In a long-term EAE mouse model, Li and colleagues demonstrated that ALA exerted significant neuroprotective, anti-inflammatory, and antioxidant effects, as evidenced by reduced clinical severity, diminished demyelination and axonal injury, downregulation of TNF-α, increased TGF-β and Treg levels, and attenuation of oxidative stress markers, supporting its potential as an adjunct immunometabolic therapy in progressive multiple sclerosis [86]. Similarly, in focal cortical EAE models, ALA reduced CD4^+^ T-cell and galectin-3^+^ immune cell infiltration into cortical lesions compared with vehicle-treated controls and decreased the proportion of galectin-3^+^ cells lacking projections, suggesting reduced monocyte recruitment [87]. Collectively, these findings underscore ALA’s multi-targeted antioxidant, anti-inflammatory, and immunomodulatory actions in EAE, supporting translational relevance for MS while highlighting the need for mechanistic and dose–response studies.

Experimental studies provide preliminary evidence that biotin may exert direct effects on oligodendrocyte lineage cells relevant to myelin repair in MS. In vitro, pharmacological biotin enhanced cell viability, promoted myelin-like ensheathment in a nanofiber assay, and increased ATP production [50]. High-dose pharmaceutical-grade biotin (MD1003) further improved progenitor survival under metabolic stress, promoted differentiation of murine and human oligodendrocyte progenitor cells, and accelerated ensheathment in vivo, supporting its role in sustaining axon–glia metabolic coupling [88]. A more recent study using a novel biotin derivative demonstrated dose-dependent neuroprotective effects in animal models, including increased expression of synaptic transmission proteins such as Synapsin-1, PSD-93, and PSD-95 [89]. Overall, while these preclinical findings suggest potential roles for biotin in oligodendrocyte differentiation and metabolism, the evidence remains limited, model systems vary, and definitive effects on remyelination have yet to be established.

#### 3.4.4. Amyotrophic Lateral Sclerosis

Amyotrophic lateral sclerosis (ALS) is characterized by progressive motor neuron degeneration associated with oxidative stress, impaired energy metabolism, and mitochondrial dysfunction [2,4]. In SOD1 transgenic ALS mice, dietary administration of ALA produced a significant extension of survival, suggesting that redox modulation may influence disease outcome. Although functional and cellular endpoints were not assessed, this survival benefit supports further exploration of ALA as a potential therapeutic agent [90]. Subsequent studies using NSC-34 motor neuron-like cells overexpressing mutant hSOD1, as well as *Drosophila* models expressing SOD1^G85R or SOD1^G93A, reported consistent reductions in reactive oxygen species, preservation of endogenous antioxidant defenses, and improvements in cell viability and locomotor performance. These effects were linked to activation of PI3K/Akt and ERK signaling pathways, as pharmacological inhibition of these pathways attenuated the observed neuroprotection [91]. Recent reviews of preclinical and translational evidence further reinforce the biological plausibility of ALA in ALS and related motor neuron disorders, highlighting convergent antioxidant, mitochondrial, and anti-inflammatory actions that mitigate oxidative and metabolic stress in vulnerable motor neurons. Although controlled clinical evidence remains limited, the mechanistic consistency across experimental systems supports its potential as a multitarget adjunctive strategy [92]. Collectively, available preclinical data position this compound as a molecule of interest in ALS research, acting through redox regulation and activation of pro-survival signaling pathways. However, the translational strength of these findings is limited by the predominance of familial SOD1 models and the scarcity of recent rodent studies. Expanded investigations, particularly in sporadic ALS models and under rigorous dosing paradigms, are required to clarify its therapeutic potential.

In comparison, preclinical evidence for biotin in ALS remains limited, with most data derived from mechanistic and indirect models rather than ALS-specific efficacy studies. Although a patent describes the design and rationale for high-dose biotin administration in SOD1-G93A transgenic mice, aiming to improve neuronal energy metabolism and delay disease progression, peer-reviewed studies confirming such effects remain unavailable. The patent outlines a therapeutic strategy based on biotin’s role as a coenzyme for carboxylases involved in anaplerosis and lipid metabolism, proposing that enhanced mitochondrial function might support motor neuron survival in ALS. However, no peer-reviewed studies have yet confirmed benefits in survival, motor performance, or neuropathology, revealing a clear gap between theoretical rationale and experimental validation [93]. Nonetheless, the absence of rigorous ALS rodent studies underscores a major translational gap, and future investigations must validate dosing, pharmacokinetics, and efficacy across diverse ALS models before clinical application can be substantiated.

#### 3.4.5. Huntington’s Disease

Huntington’s disease (HD) is an autosomal dominant neurodegenerative disorder caused by an expanded CAG repeat in the HTT gene, producing mutant huntingtin protein that drives neuronal loss in key brain regions and results in progressive motor, cognitive, and psychiatric impairments [2,4]. Preclinical studies examining ALA in HD remain limited but offer promising insights. In transgenic mouse models (R6/2, N171-82Q), treatment prolonged survival and attenuated disease progression, suggesting that redox modulation can influence HD pathophysiology [94]. In toxin-induced HD models using 3-nitropropionic acid, co-administration with acetyl-L-carnitine reduced mitochondrial swelling, improved cognitive performance, and reversed bioenergetic deficits, highlighting the capacity of mitochondrial modulators to preserve organelle integrity under conditions of oxidative stress [95].

These observations highlight ALA’s ability to restore redox balance and protect neuronal macromolecules from oxidative injury, processes that are similarly compromised in HD and other neurodegenerative conditions. Although such effects have not been extensively validated in HD-specific models, they align with current evidence implicating mitochondrial dysfunction, oxidative damage, and impaired endogenous antioxidant defenses as central components of HD pathology [96]. Collectively, these observations support the mechanistic plausibility of ALA as a neuroprotective agent in HD. However, the available evidence remains limited, with variability in model systems, dosing regimens, and endpoints, constraining direct comparisons and reducing translational confidence.

Recent preclinical evidence highlights the potential of thiamine and biotin supplementation in HD. Lim et al. [97] reported that oligodendrocyte maturation arrest in R6/2 mice and human HD brains is linked to reduced thiamine pyrophosphokinase 1 expression, and that combined vitamin supplementation in R6/1 mice restored oligodendrocyte differentiation, corrected myelin-related transcriptional dysregulation, and attenuated neuronal pathology. These findings suggest that targeting vitamin-dependent metabolic pathways may help restore bioenergetic and glial homeostasis in HD [97]. Taken together, these findings underscore the mechanistic importance of targeting metabolic dysfunction in HD, although clinical efficacy remains untested. Notably, no studies have examined biotin alone in HD models; current insights into its neuroprotective role derive from other neurological and metabolic contexts. Given biotin’s established role in energy metabolism, further animal studies are needed to assess its capacity to reduce motor deficits, neuronal loss, and mitochondrial dysfunction in HD.

A comprehensive overview of the key findings across these conditions is provided in the following section (Table 1). 

While mechanistic and experimental studies provide a biological rationale for considering ALA and biotin as potential neuroprotective candidates, their translational relevance ultimately depends on whether these effects can be observed in human disease. Accordingly, the following section reviews the available clinical evidence and examines the extent to which mechanistic and experimental findings have been translated into measurable outcomes in patients.

### 3.5. Clinical Evidence and Translational Perspectives

Clinical evidence for ALA is more mature than for biotin. Small-scale trials of ALA have explored Alzheimer’s disease and multiple sclerosis, with some signals of potential efficacy, while biotin trials have largely focused on progressive multiple sclerosis, with mixed and inconclusive results. This imbalance highlights the need for clinical trials evaluating biotin beyond MS, as well as larger, rigorously designed studies on ALA to determine whether preclinical findings translate into clinically meaningful benefit.

#### 3.5.1. Alzheimer’s Disease

Clinical investigations of ALA in AD remain limited and heterogeneous. An early open-label longitudinal study administered 600 mg/day ALA to 43 AD patients for up to 48 months in addition to standard cholinesterase inhibitors, reporting a markedly slower rate of cognitive decline compared with expected trajectories in untreated cohorts, particularly in patients with milder disease; however, the absence of randomization and placebo control substantially limits interpretation [98]. More rigorously designed but smaller trials have evaluated ALA in combination with omega-3 fatty acids. In a 12-month randomized, placebo-controlled pilot study, the combination (ω-3 + ALA) slowed decline in MMSE scores (*p* < 0.01) and Instrumental Activities of Daily Living (*p* = 0.01) compared with placebo, although no significant differences were observed in oxidative stress markers or on the ADAS-cog scale [99]. Preliminary clinical observations suggest that ALA may offer cognitive and metabolic benefits in AD patients with insulin resistance. In a small exploratory study, ALA supplementation produced modest improvements in cognitive function and metabolic control, consistent with its antioxidant and insulin-sensitizing properties. However, limited sample size and study duration warrant cautious interpretation and further validation in larger controlled trials [100].

Other early clinical studies administering 600 mg/day ALA to patients with AD or related dementias alongside standard therapies also suggested cognitive benefit, though these studies were limited by small cohorts, lack of randomization, and short follow-up [101]. Furthermore, in a 16-week randomized trial, Galasko et al. [102] evaluated a combination of vitamin E, vitamin C, and ALA in mild-to-moderate AD. The intervention significantly reduced cerebrospinal fluid F_2_-isoprostanes, indicating decreased oxidative stress; however, it produced no changes in tau or Aβ_42_ biomarkers and was unexpectedly associated with a faster cognitive decline on Mini-Mental State Examination (MMSE) scores [102]. Overall, current clinical evidence for ALA in AD remains inconsistent, with signals of possible cognitive or biomarker benefit but data that are insufficiently robust for firm conclusions. Larger, well-controlled trials are required to clarify its therapeutic value and establish clinical relevance.

Despite compelling mechanistic rationale from preclinical studies, there is no clinical trial evidence to support biotin as an AD therapy at present. Indirect human evidence suggests a possible link between biotin status and dementia risk. A large prospective analysis of the UK Biobank (*n* ≈ 122,959) reported that higher dietary biotin intake was associated with a reduced risk of all-cause dementia, including Alzheimer’s disease, although the observational design precludes causal inference [103]. Additionally, conference abstract data reported biotin deficiency and abnormal pantothenic acid levels in dementia patients, pointing to potential metabolic disruptions, though the non-peer-reviewed nature of these findings warrants cautious interpretation (P3-400, 2006) [104]. At present, available evidence is restricted to epidemiological associations and mechanistic observations, which highlight the need for rigorously designed human studies to clarify whether biotin can exert meaningful effects on cognition, functional outcomes, or disease biomarkers in Alzheimer’s disease.

#### 3.5.2. Parkinson’s Disease

Clinical evaluation of ALA in Parkinson’s disease (PD) remains limited. As highlighted by Rabin et al. [105], current support for its use is largely theoretical, relying predominantly on preclinical data and anecdotal observations, with no randomized controlled trials to date demonstrating clear symptomatic or disease-modifying efficacy. Despite its antioxidant rationale and favorable safety profile, the absence of standardized data underscores the need for rigorously designed studies to clarify its therapeutic relevance in PD [105]. Existing evidence is confined to small, open-label, non-randomized, and primarily safety-focused investigations. As summarized by Przewodowska et al. [106], exploratory human evaluation of ALA combined with acetyl-L-carnitine in progressive supranuclear palsy reported acceptable tolerability; however, published data remain insufficient to determine whether this approach provides clinically meaningful benefits on motor or functional outcomes [107]. Reviews also emphasize the lack of controlled clinical trials of ALA in PD. The only registered human study relevant to parkinsonian syndromes (ClinicalTrials.gov identifier NCT01537549) evaluated the safety and tolerability of ALA in combination with acetyl-L-carnitine in patients with progressive supranuclear palsy (PSP), an atypical parkinsonian disorder rather than idiopathic PD; however, no peer-reviewed results or outcome data from this study have been published [107]. This early-phase open-label trial appears to have been designed primarily to assess feasibility and safety rather than therapeutic efficacy, which limits the interpretation of its clinical relevance. In summary, although ALA remains a mechanistically plausible antioxidant candidate for PD, current clinical evidence is insufficient to support therapeutic benefit. Future well-controlled trials are required to determine whether ALA may have a role as an adjunctive or disease-modifying intervention.

To date, no clinical trials have examined biotin supplementation in PD. Nevertheless, emerging microbiome-based evidence suggests potential relevance. A recent meta-analysis of shotgun metagenomic data by Nishiwaki et al. [108] identified reduced gut microbial capacity for biotin biosynthesis in PD patients, indicating altered vitamin-producing pathways within the gut–brain axis. Given biotin’s essential role in mitochondrial and lipid metabolism, such deficiencies may contribute to metabolic stress and neurodegenerative processes. These findings highlight the need to explore whether restoring biotin availability could influence PD-related metabolic and inflammatory mechanisms [108]. Overall, while biotin-related pathways appear biologically significant, its therapeutic efficacy in PD remains untested in humans, underscoring the need for well-designed clinical trials.

#### 3.5.3. Multiple Sclerosis

Recent clinical evidence suggests potential benefits of ALA in multiple sclerosis (MS), particularly in secondary progressive MS (SPMS). A recent meta-analysis of randomized controlled trials reported significant improvements in disability-related outcomes, including physical function and Expanded Disability Status Scale (EDSS) scores. These effects are broadly consistent with its antioxidative and mitochondrial-supportive mechanisms. Nevertheless, the authors highlighted important limitations, including small sample sizes, study heterogeneity, and incomplete reporting of complementary outcomes, underscoring the need for larger and longer-term trials [109].

Additional clinical studies not included in the meta-analysis further inform its translational profile. In a 14 day pilot trial, Yadav et al. [110] reported good tolerability and dose-dependent serum exposure in patients with MS, accompanied by reductions in matrix metalloproteinase-9 (MMP-9) and soluble intercellular adhesion molecule-1 (sICAM-1), biomarkers linked to T-cell migration and disease activity [110]. In a subsequent 2 year randomized, double-blind pilot study in SPMS, daily oral administration (1200 mg) was associated with modest improvements in walking performance—specifically faster turning time in the Timed Up and Go test—among participants with lower baseline disability, although balance measures were unchanged [111]. Finally, in both healthy controls and SPMS subjects, short-term oral supplementation (1200 mg/day for two weeks) increased intracellular cAMP levels in peripheral blood mononuclear cells, supporting a dose-dependent immunomodulatory mechanism relevant to MS pathophysiology [112]. Taken together, these findings suggest a potential benefit of ALA in MS, while underscoring the need for larger, multicenter Randomized Controlled Trials (RCTs) with broader clinical and biomarker endpoints.

The most notable clinical application of high-dose biotin has been in progressive multiple sclerosis. An initial open-label pilot study reported that high-dose biotin (100–300 mg/day) improved motor disability and, in some cases, visual function in patients with progressive MS. These effects were attributed to enhanced myelin synthesis and metabolic support, though confirmation in controlled trials remains necessary [113]. This was followed by the multicenter, randomized, double-blind, placebo-controlled MS-SPI study (*n* = 154), which showed that high-dose biotin (MD1003, 300 mg/day) led to significant improvements in disability and walking speed in approximately 13% of participants, compared with none in the placebo group. These findings provided the first controlled evidence supporting the therapeutic potential of biotin in progressive MS [114]. In contrast, an open-label series administering high-dose biotin (~300 mg/day for up to 12 months) found the treatment to be safe and well tolerated but without sustained clinical benefit; in fact, more than one-third of patients worsened during therapy, suggesting limited efficacy and raising the possibility that disease progression or impaired metabolic capacity of damaged CNS tissue accounted for outcomes [114].

Additional exploratory work includes the MS-ON study, a randomized, double-blind, placebo-controlled study evaluating high-dose biotin (MD1003) in patients with chronic visual loss due to optic neuritis associated with MS. The study found that the treatment was generally well tolerated and produced modest trends toward improvement in visual evoked potentials and visual acuity; however, these results did not reach statistical significance. The study underscored both the potential and the challenges of translating biotin’s metabolic mechanisms into measurable functional recovery within demyelinating optic pathways [115]. More conclusively, the phase 3 SPI2 trial, a large multicenter, randomized, double-blind, placebo-controlled study, evaluated high-dose biotin (MD1003) in 642 patients with MS. Cree et al. [116] found no significant differences between treatment and placebo groups in EDSS scores or walking speed over 108 weeks. The study also reported similar rates of adverse events across groups, confirming the compound’s safety but not confirming earlier efficacy signals from smaller studies [116]. A systematic review and meta-analysis pooling three RCTs (*n* = 889, mostly progressive MS) testing high-dose biotin (≥300 mg/day for 12–15 months) as add-on therapy concluded that biotin did not improve disability or EDSS, although a modest effect on walking speed (Timed 25-Foot Walk) was observed; overall adverse events were comparable to placebo, with the important caveat of laboratory test interference [117]. In a related context, a phase 2b open-label pilot study evaluated high-dose pharmaceutical-grade biotin (hdPB) in patients with chronic demyelinating peripheral neuropathies, including CIDP, anti-MAG neuropathy, and Charcot–Marie–Tooth disease 1A/1B. Although not directly focused on MS, the investigation was conceptually linked through shared demyelinating mechanisms. Treatment with hdPB improved sensory and motor parameters, nerve excitability, and gait performance, suggesting a potential role for biotin in enhancing remyelination and axonal function in peripheral nervous system disorders with pathophysiological parallels to MS [118]. This translational gap is particularly evident in progressive multiple sclerosis. Although high-dose biotin was initially supported by a biologically plausible metabolic rationale and encouraging early clinical signals, the large phase III SPI2 trial failed to confirm a significant benefit on disability improvement or walking-related outcomes. These findings underscore the limitations of extrapolating from mechanistic plausibility and selected preclinical models to complex human disease. They also suggest that disease-stage heterogeneity, endpoint selection, and incomplete understanding of CNS target engagement may have contributed to the discrepancy between early expectations and later confirmatory evidence. Accordingly, high-dose biotin should be interpreted with caution as a biologically interesting but clinically unconfirmed strategy in progressive MS.

Taken together, clinical investigations of high-dose biotin in progressive MS have yielded mixed and largely inconclusive results. While the biological rationale for biotin remains compelling, current evidence does not support its therapeutic use outside controlled research settings, underscoring the urgent need for larger, well-powered trials to clarify its role.

#### 3.5.4. Amyotrophic Lateral Sclerosis

Clinical evidence evaluating ALA in amyotrophic lateral sclerosis (ALS) remains extremely limited. To date, no randomized controlled trials have demonstrated efficacy on motor decline or survival. The most comprehensive appraisal is provided by ALSUntangled #79, which reviewed the available literature and identified only anecdotal patient reports, small open-label observations, and mechanistic plausibility, without rigorous trial evidence supporting clinical benefit [92]. Registry data further underscore this gap. An interventional clinical trial (NCT04518540) was registered to assess safety, tolerability, and preliminary efficacy as an adjunctive antioxidant therapy in patients with ALS; however, as of the most recent update, no peer-reviewed results or published outcomes have been reported [119]. Although ALA is biologically plausible as a neuroprotective compound, current human evidence remains confined to anecdotal or preliminary observations. Well-designed, adequately powered clinical trials are required to determine whether this approach can translate into a viable therapeutic option for ALS.

High-dose biotin has been evaluated clinically in ALS. In a Phase 2 randomized, double-blind, placebo-controlled pilot trial, MD1003 (300 mg/day) was administered to 30 ALS patients (20 receiving treatment and 10 placebo) and followed over 24 weeks. The intervention was found to be safe and well tolerated; however, no significant improvement in motor function, as assessed by the ALS Functional Rating Scale–Revised (ALSFRS-R), was observed compared with placebo (*p* = 0.49) [120]. There is also a completed clinical trial registered (NCT03427086) that evaluated safety and tolerability of high-dose biotin (300 mg/day) over six months, with secondary endpoints including motor disability (ALS-FRS), respiratory function, and weight change, but results have not been published in peer-reviewed form yet [121]. Clinical evidence of biotin in ALS so far demonstrates tolerability and a favorable safety profile, but there is no current evidence showing efficacy in altering disease progression or improving motor outcomes. Larger, well-designed trials with published results are needed to assess biotin’s therapeutic potential in ALS.

#### 3.5.5. Huntington’s Disease

Published clinical evidence for ALA in Huntington’s disease is extremely limited, with no randomized trials evaluating α-lipoic acid as a standalone intervention. Notably, the randomized human study sometimes mislinked to ALA in this setting tested highly unsaturated fatty acids (HUFAs), not α-lipoic acid; moreover, in the fatty-acid literature the acronym “ALA” commonly denotes α-linolenic acid, underscoring the risk of misattribution [122]. In conclusion, while preclinical findings justify interest in ALA for HD, its therapeutic relevance in patients remains untested, and rigorously designed clinical trials are essential to clarify its potential.

Clinical research on biotin in HD is currently limited to a single ongoing Phase 2 trial (HUNTIAM, NCT04478734). This randomized study is designed to evaluate the safety, tolerability, and biological effects of combined thiamine and biotin therapy in patients with mild to moderate HD over one year, with primary biomarker outcomes including thiamine monophosphate levels in cerebrospinal fluid. However, as of September 2025, no peer-reviewed results have been published, leaving the clinical efficacy of biotin in HD unproven [123]. It is important to emphasize that current clinical evidence is restricted to a registered trial without available results. This highlights a translational gap between promising preclinical findings and human validation, underscoring the need for caution when interpreting the therapeutic potential of biotin in HD until robust trial data become accessible.

A comprehensive overview of the available clinical evidence across these conditions is presented below (Table 2).

### 3.6. Safety, Pharmacokinetics, and Limitations Regarding the Use of Alpha-Lipoic Acid and Biotin

#### 3.6.1. Alpha-Lipoic Acid

Human and in vitro studies indicate that intestinal uptake of ALA is carrier-mediated, pH- and energy-dependent, with evidence implicating MCT and possibly SMVT [12,14]. Human pharmacokinetic investigations indicate that oral ALA, typically administered at 300–600 mg, is rapidly absorbed, achieving Tmax values of 30–60 min and exhibiting low but variable oral bioavailability in the 20–40% range due to extensive first-pass hepatic metabolism. Teichert et al. demonstrated a short elimination half-life (~30 min) and minimal urinary excretion of the parent compound, whereas Hermann et al. further revealed a substantial food effect reducing systemic exposure and marked enantioselective differences, particularly higher R-ALA bioavailability, underscoring the importance of fasting administration and highlighting clinically relevant interindividual variability [125,126].

Evidence for blood–brain barrier (BBB) penetration of ALA and DHLA remains mixed and appears to depend on dose, route, and analytical methodology. Animal studies report measurable brain exposure after parenteral administration, whereas rigorous LC–MS/MS analyses show negligible brain levels following standard oral dosing once vascular contamination is controlled [127]. While contemporary reviews frequently cite BBB permeation based on older animal data, definitive human pharmacokinetic of BBB penetration remains unavailable [14].

Across randomized and long-term clinical studies, ALA has generally demonstrated a favorable safety profile at therapeutic doses, most commonly 600 mg/day orally (for weeks to years) or 600 mg/day intravenously for 2–4 weeks, with adverse events largely mild and dose-related, primarily gastrointestinal symptoms and vertigo. No consistent excess of serious adverse events has been reported compared with placebo, and similar tolerability has been observed at 1200 mg/day in secondary progressive multiple sclerosis [128]. A two-year RCT in secondary progressive MS (1200 mg/day) likewise confirmed acceptable tolerability and reported a reduction in brain atrophy, supporting clinical safety beyond diabetic neuropathy populations [124]. Meta-analytical evidence from a placebo-controlled studies indicates that ALA significantly improves neuropathic symptoms and sensory deficits, while higher doses (1200–1800 mg/day) produced no additional benefit and increased gastrointestinal adverse events. These findings contributed to the adoption of 600 mg/day as the reference therapeutic dose used in subsequent clinical and pharmacological evaluations [129].

Hepatotoxicity has not been attributed to ALA in routine clinical use, with the NIH LiverTox monograph classifying it as an unlikely cause of liver injury (“E” likelihood). Nevertheless, isolated case reports of acute liver injury underscore the importance of continued pharmacovigilance [130]. Safety assessments have also identified rare cases of insulin autoimmune syndrome (IAS), typically presenting hypoglycemia in genetically predisposed individuals. In this context, the EFSA Panel on Nutrition, Novel Foods and Food Allergens (2021) concluded that ALA supplementation may increase the risk of IAS, particularly among carriers of specific HLA alleles, although the condition generally resolves after discontinuation and no consistent evidence of broader systemic or pediatric toxicity has been reported [131]. Consistent with these regulatory assessments, Capece et al. [29] highlighted an association with IAS in susceptible individuals, supporting the need for glucose monitoring when this intervention is combined with glucose-lowering therapies, given its potential to influence insulin dynamics [29]. In sum, clinical evidence supports low intrinsic toxicity and good tolerability of ALA at standard doses. Prudent clinical use entails fasting administration to optimize absorption and vigilance for rare but serious risks such as IAS-related hypoglycemia and potential overdose.

#### 3.6.2. Biotin

Biotin is absorbed in the small intestine primarily through the SMVT and, at higher concentrations, partially via passive diffusion. The SMVT also facilitates cellular uptake across several tissues, including the blood–brain barrier [132,133]. Following oral administration, biotin is rapidly absorbed in most individuals, reaching peak plasma concentrations within 1–2 h. Circulating levels vary substantially depending on the supplementation dose, as pharmacological intake can greatly exceed physiological concentrations and interfere with immunoassays, requiring up to 72 h of discontinuation prior to testing, thereby highlighting the clinical relevance of its pharmacokinetic properties [134,135]. Recommended washout intervals may vary depending on the specific assay platform, biotin dose, and renal function; therefore, clinicians and laboratories should follow manufacturer and regulatory guidance and consider the use of biotin-resistant immunoassays when available. In a PET imaging study in rodents, intravenously administered [^11C]-biotin displayed rapid distribution to the liver, kidneys, brain, retina, and heart, with evidence of dose-dependent urinary excretion and transporter-mediated uptake in SMVT-expressing tissues, providing in vivo insight into biotin trafficking and renal clearance dynamics. Supplementation markedly increases systemic exposure: a 10 mg dose raises plasma concentrations to approximately 55–140 ng/mL, whereas 100 mg results in 375–450 ng/mL [136]. At pharmacological doses of 100–300 mg/day, plasma levels remain elevated due to saturable transport and slower clearance, leading to accumulation and attainment of steady-state within about three days. Biotin elimination is dose-dependent, with an estimated half-life of ~2 h under nutritional conditions and extending to nearly 19 h at higher doses. Although biotin is cleared relatively rapidly in healthy individuals, plasma concentrations decline to <30 ng/mL within 3.5–8 h after a 5–10 mg dose; elevated levels may persist for several days following high-dose intake, particularly in those with renal impairment [114,134].

Dietary biotin is generally well tolerated, and even high-dose regimens used in multiple sclerosis (e.g., 300 mg/day) have demonstrated acceptable safety profiles in clinical trials, with gastrointestinal symptoms and headache among the most frequent but generally mild adverse events [35,116].

The principal safety concern associated with high-dose biotin is not classical organ toxicity but rather analytical interference in immunoassays that rely on streptavidin–biotin capture systems. Elevated circulating biotin can generate falsely low values in sandwich assays and falsely high values in competitive assays, thereby affecting clinically relevant biomarkers such as thyroid hormones, thyroid-stimulating hormone, and cardiac troponins, potentially leading to diagnostic confusion or inappropriate clinical management [134]. This issue is particularly relevant in both routine clinical practice and in clinical trials, where misinterpretation of laboratory results or trial endpoints may compromise clinical decision-making or the accurate assessment of treatment-related effects. For this reason, recent biotin intake should be actively considered when interpreting laboratory results, and the use of biotin-resistant assay platforms or temporary discontinuation before sampling should be considered when appropriate, as recommended by regulatory agencies and laboratory guidelines [135].

Beyond assay interference, serious toxicity is rare, even at doses of 10–50 mg/day and in patients receiving up to ~200 mg/day orally or 20 mg/day intravenously for biotinidase deficiency, supporting the absence of a defined upper intake level [137]. Clinical trials with pharmacological biotin have generally reported adverse-event rates similar to placebo, aside from more frequent assay-related laboratory anomalies [117,134,135]. Additionally, animal data indicates that high-dose biotin supplementation may induce morphological changes in hepatic tissue without concomitant elevations in conventional toxicity biomarkers, underscoring that although overt clinical toxicity remains rare, subclinical alterations may occur at pharmacological levels [138]. Moreover, in an animal model, pharmacological biotin supplementation induced transcriptional changes in CYP1A1/1A2 expression without corresponding alterations in enzymatic activity or protein levels, suggesting minimal impact on xenobiotic metabolism despite high-dose exposure [139]. In sum, particular vigilance is warranted in populations with polypharmacy or renal impairment, where accumulation may occur and produce marked spurious shifts in endocrine test results, which normalize upon discontinuation.

## 4. Discussion

Taken together, the clinical literature suggests that biological plausibility alone is insufficient to ensure therapeutic efficacy, making it necessary to interpret these findings within a broader translational framework that considers pharmacokinetics, disease heterogeneity, endpoint selection, and biomarker sensitivity.

For ALA, interindividual variability in bioavailability is better explained by limited endogenous mitochondrial synthesis, low and heterogeneous dietary intake, and pharmacokinetic constraints such as rapid clearance and variable absorption, rather than by consistent exogenous supply. Together, these features pose formulation and adherence challenges that may limit sustained CNS exposure and therapeutic efficacy. Accordingly, the development of standardized formulations—such as controlled-release preparations or R-enantiomer-enriched formulations—warrants prioritization. In contrast, for biotin, assay interference remains a distinct translational challenge, complicating safety monitoring and potentially confounding clinical trial endpoints that rely on laboratory biomarkers. In addition, consensus is needed regarding optimal dosing regimens, as clinical investigations to date have employed a wide range of doses. More broadly, and consistent with the nutraceuticals literature, dose-dependent toxicities, variable bioavailability, and drug–nutraceutical interactions may emerge, particularly with prolonged use or in polypharmacy settings, underscoring the need for harmonized quality standards, prespecified interaction assessments, and embedded pharmacovigilance in trial design. Recent analyses emphasize that adverse event reporting for nutraceuticals remains inconsistent and underdeveloped, limiting recognition of organ-specific toxicities and drug interactions [140]. Regulatory heterogeneity across markets further complicates labeling and safety oversight [141], reinforcing the case for standardized reporting and risk-mitigation frameworks in ALA and biotin studies.

A recurring challenge in the clinical translation of both ALA and biotin is that encouraging mechanistic and preclinical findings have not consistently translated into meaningful clinical benefit. Several factors may account for this discrepancy. First, neurodegenerative diseases are biologically heterogeneous and are often diagnosed after substantial tissue damage has already occurred, which may limit the impact of metabolic or redox-targeted interventions. Second, differences in formulation, bioavailability, CNS penetration, dosing schedules, and treatment duration may substantially influence pharmacodynamic exposure in humans. Third, some clinical endpoints may be insufficiently sensitive to detect modest biological effects, particularly in slowly progressive disorders. Finally, interventions that act on broad metabolic and inflammatory pathways may be more effective as adjunctive or stage-specific strategies than as standalone therapies. These considerations may help explain why clinically promising signals have remained inconsistent despite strong biological plausibility.

Additionally, disease heterogeneity and complexity, suboptimal endpoint selection, and the paucity of validated pharmacodynamic biomarkers further constrain interpretation. Future research will require well-designed, large-scale randomized multicenter trials with robust methodology to clarify efficacy in neurodegenerative diseases such as AD, PD, MS, ALS, and HD. These studies should include well-defined inclusion criteria, disease-stage stratification, and longer follow-up periods to detect clinically meaningful changes in progression rates. Incorporating preplanned safety monitoring for potential pro-oxidant effects and organ-specific toxicities reported with certain nutraceuticals will be essential to balance benefit and risk. Indeed, recent systematic evaluations have shown that hepatotoxicity, renal impairment, and cardiovascular complications have been linked to specific dietary supplements, stressing the importance of proactive risk surveillance in future trials [140,141].

The absence of reliable biomarkers for treatment response has limited progress. Emerging candidates include neurofilament light chain (NfL), a blood and CSF marker of neuroaxonal injury, is increasingly used for prognostication and monitoring in MS and other neurological disorders [141]. Measures of oxidative stress, particularly F2-isoprostanes and redox status (e.g., brain or CSF glutathione via MR spectroscopy or biochemical assays), are also gaining traction, with recent studies linking these markers to AD pathophysiology and cognitive outcomes [142,143]. Neuroimaging endpoints such as MRI-based brain atrophy rates and myelin integrity metrics (e.g., myelin water fraction, g-ratio/microstructural mapping) provide quantitative readouts that can track disease progression and treatment effects [144]. Incorporating such biomarkers into future trials will be critical for pharmacodynamic validation and patient stratification.

Given the multifactorial nature of neurodegenerative disorders, biotin and ALA are unlikely to be curative as monotherapies. Their future may lie in combination approaches: either together, to simultaneously address mitochondrial metabolism (biotin) and oxidative/inflammatory stress (ALA); or in conjunction with disease-modifying therapies such as cholinesterase inhibitors (in AD), dopaminergic drugs (in PD), or immunomodulators (in MS). Because nutraceuticals may also amplify or inhibit drug effects via shared pathways, combination regimens should include prospective interaction screening and therapeutic drug monitoring plans where appropriate [141]. In this context, nutrivigilance frameworks have been proposed to systematically capture real-world adverse events in nutraceutical users, representing a necessary complement to pre-approval clinical data [145].

Not all patients may benefit equally from biotin or ALA. Personalized strategies that account for genetic background, metabolic profile, disease stage, and comorbidities are needed. Advances in omics technologies (genomics, metabolomics, lipidomics) may identify patient subgroups more likely to respond, facilitating precision medicine in neurodegeneration. In parallel, advances in formulation science, including nanotechnology-enabled delivery systems, may improve bioavailability while reducing toxicity risk, aligning with an emerging precision-dosing paradigm for nutraceuticals [141].

## 5. Conclusions

Neurodegenerative disorders continue to pose a major challenge to modern medicine, with disease-modifying therapeutic options remaining limited. This narrative review highlights the complementary and potentially additive actions of ALA and biotin on key molecular pathways involved in neurodegeneration, particularly those regulating mitochondrial function, oxidative balance, redox-sensitive signaling cascades, and inflammatory processes. An integrative analysis of preclinical data together with limited clinical evidence suggests that these compounds may modulate neuronal resilience and energy homeostasis through overlapping mechanisms, including activation of the NRF2–AMPK–cGMP–PKG-Akt axis.

Overall, ALA is supported by a relatively broad and consistent preclinical literature across multiple neurodegenerative disease models, whereas biotin has undergone more extensive clinical evaluation primarily in progressive multiple sclerosis, with heterogeneous outcomes. Outside MS, clinical data for biotin remain sparse and largely exploratory. Both compounds converge on mitochondrial, redox, and inflammatory pathways relevant to neurodegeneration and exhibit generally favorable safety profiles. While current evidence supports their biological plausibility as adjunctive or exploratory therapeutic strategies, their clinical efficacy, optimal dosing, and disease-specific applicability remain to be clearly established.

In this context, the proposed ALA–biotin framework should not be interpreted as a definitive therapeutic strategy but rather as a biologically grounded hypothesis that warrants systematic experimental validation. Its value lies in integrating metabolic, redox, and signaling dimensions into a coherent model that may guide future translational research.

Despite the mechanistic share common nodes identified across preclinical models, several key uncertainties remain unresolved. Direct evidence evaluating the combined use of ALA and biotin in CNS disease models is still scarce, and the extent to which their effects are additive, complementary, or context-dependent is still unclear. Future studies should prioritize combination-oriented designs, pharmacodynamic biomarkers of target engagement, improved characterization of CNS exposure, and disease-specific clinical endpoints capable of capturing early biological responses. Clarifying these issues will be essential before these compounds can be positioned more confidently within translational neurotherapeutic strategies.

## Figures and Tables

**Table 1:** Preclinical and Mechanistic Evidence of Alpha-Lipoic Acid and Biotin in Neurodegenerative Disease Models.

Compound/Model	Design/Dose/Duration	Primary Outcome/Mechanism	Ref.
Alpha-Lipoic Acid (ALA)			
Preclinical in vivo studies (Neurodegenerative models)			
In vitro human immune cells (T cell-enriched PBMCs, NK cells) + ex vivo PBMCs from multiple sclerosis (MS) subjects	In vitro: ALA 10–100 µg/mL, short pretreatment (1–5 min), immune stimulation 6–24 h. In vivo: (MS): Oral ALA 1200 mg, single dose; PBMCs analyzed at 4 h	↓ IL-6 and IL-17 secretion in T cell-enriched PBMCs; ↓ T-cell activation (IL-2) and proliferation; ↑ cAMP → ↑ PKA signaling (PKA inhibition blocks these effects)	[20]
Dapsone-treated mouse model (prefrontal cortex and hippocampus)	ALA administered at 25 mg/kg → co-treatment with DDS over chronic exposure in mice	↓ Microglial and astrocytic activation; ↓ pro-inflammatory cytokines (IL-1β, IL-17, IL-4); ↑ BDNF; ↑ antioxidant capacity (TEAC, GSH, SOD, CAT) → ↓ oxidative damage and iron accumulation in PFC/hippocampus	[21]
Fischer 344 rats (young, middle, old)—brain aging model	In vivo; Fischer 344 rats of 6, 12, 24 months; ALA given (dose ~100 mg/kg diet or similar) over aging period (weeks/months)	↑ Brain glucose uptake (µPET); restoration of Akt–JNK signaling balance; ↑ PGC-1α-mediated mitochondrial biogenesis → improved mitochondrial bioenergetics and energy metabolism	[22]
APP23/PS45 transgenic mice (Alzheimer’s model)	In vivo; male APP23/PS45 mice, ALA 5 mg/kg/day orally for 4 months starting at 2 months of age	↑ Mitophagy (BNIP3L-mediated) → ↑ ADAM10 α-secretase cleavage of APP → ↓ amyloid-β plaques; ↑ cognitive performance in behavioral tests	[23]
Rotenone—induced Parkinson’s disease in mice	In vivo; male mice (*n* ≈ 40) divided into four groups: control; ALA 100 mg/kg/day i.p.; rotenone 1.5 mg/kg i.p. every 2 days; rotenone + ALA; duration: 21 days	Activation of the PI3K–Akt pathway; ↓ caspase-3 activation; ↓ IL-1β, TNF-α, and NF-κB; ↑ antioxidant markers (GSH, SOD); ↓ MDA → improved behavioral and motor outcomes	[27]
Aged Tg2576 transgenic mice (Alzheimer’s model)	In vivo; 10-month-old Tg2576 and wild-type mice; diet containing 0.1% ALA (~100 mg/kg/day) for 6 months	↑ Hippocampal-dependent learning and memory (Morris water maze, contextual fear paradigm); ↓ nitrotyrosine (nitrative stress marker); no change in Aβ levels → neuroprotection via reduction of nitrative and oxidative stress	[71]
3 × Tg-AD mice (Alzheimer’s model)	In vivo; Tg-AD mice; ALA 0.23% (≈60 mg/kg/day) in diet for 4 weeks	Restoration of cerebral glucose oxidation and TCA cycle flux (↑ Glu, Gln, Asp labeling); reversal of age-associated hypometabolism → improved mitochondrial bioenergetics via modulation of Akt/JNK/PGC-1α pathways	[72]
P301S transgenic mice (tauopathy/Alzheimer’s model)	In vivo; ALA 3 mg/kg and 10 mg/kg administered chronically in P301S mice (duration ~8–12 weeks)	↑ GLUT3/GLUT4; ↑ HK activity; ↑ BDNF/TrkB → ↑ HIF-1α → restored brain glucose metabolism → neuroprotection	[73]
Rat unilateral intrastriatal 6-OHDA model (Parkinsonism-like oxidative damage)	ALA 35 mg/kg, i.p., once daily for 14 days after 6-OHDA lesion (unilateral intrastriatal injection)	↓ TBARS; ↑ antioxidant defense (↑ GPx activity); ↓ motor asymmetry (trend): apomorphine-induced rotations ↓ at day 14 vs. lesion-only group, although not reaching statistical significance	[75]
Parkinson’s disease models: C57BL/6 mice (MPTP-induced) + SH-SY5Y neuroblastoma cells (MPP^+^-induced)	In vivo: ALA 50 mg/kg/day i.p., administered for 14 days; MPTP 30 mg/kg/day i.p., for 5 consecutive days; EX527 (SIRT1 inhibitor) 10 mg/kg/day i.p. (mechanistic validation). In vitro: ALA 200 µM, pretreatment 3 h before MPP^+^; MPP^+^ 1 mM, exposure 24 h; EX527 10 µM	↑ Motor performance (rotarod, pole test); ↑ Dopaminergic neuron survival (↑ TH^+^ neurons); ↑ Cell viability (SH-SY5Y); ↓ ROS production; ↑ SOD activity; ↓ MDA levels; ↑ SIRT1 and ↑ PGC-1α signaling; ↑ Mitochondrial function and oxidative stress resistance; Effects reversed by SIRT1 inhibition (EX527) → confirmation of a SIRT1-dependent PGC-1α mechanism	[76]
6-OHDA-induced PD model in Sprague—Dawley rats (unilateral intrastriatal lesion; substantia nigra endpoints). In vitro: PC12 cells exposed to 6-OHDA	In vivo: Lesion: 6-OHDA 20 μg total injected into right striatum (two sites; 10 μg/site). Treatment: ALA 100 mg/kg i.p., once daily for 14 days, initiated 4 weeks after 6-OHDA lesion (after behavioral screening). In vitro (PC12): ALA pretreatment 1 h (commonly 10 μM in key assays) → then 6-OHDA 200 μM for 24 h	↓ Motor deficits; Dopaminergic integrity preserved (prevention of TH loss); ↓ ROS (PC12); ↑ SOD and GSH in substantia nigra; ↓ iron accumulation; Iron metabolism signaling normalized: ↑ 6-OHDA-induced IRP2 and ↑ DMT1 are antagonized by ALA (rats SN + PC12) → ↑ iron homeostasis	[77]
Parkinson’s disease models (in vivo and in vitro; ferroptosis-associated neurodegeneration)	Dose: Not reported in abstract. Duration: Not reported in abstract	↓ Motor deficits (behavioral performance improved with ALA); ↓ Ferroptosis; ↑ GPX4; ↑ xCT (SLC7A11); ↓ Iron overload; ↑ FPN; ↑ FTH1; ↓ DMT1; ↓ Oxidative damage; ↓ ROS; ↓ Lipid peroxidation; ↑ SIRT1–NRF2 signaling, leading to enhanced antioxidant defense and suppression of ferroptotic cell death.	[78]
Model of Parkinson’s disease induced by (6-OHDA)	Male rats; ALA intraperitoneally (100 mg/kg or 200 mg/kg) daily for 15 days following lesion induction	Improved motor behavior (↓ apomorphine-induced rotations, ↑ locomotor activity, ↑ contralateral paw use); ↓ TBARS and nitrite levels; restoration of catalase activity	[79]
Model of Parkinson’s disease-induced dyskinesia	Male rats; ALA administered at 31.5 mg/kg (low dose) or 63 mg/kg (high dose), 3 weeks	↓ Dyskinesia; ↓ MDA, ↑ glutathione/GSH activity; ↓ Iba-1-positive cells; ↓ apoptotic markers, including cleaved caspase-3 and PARP overexpression in the substantia nigra	[80]
ALA—Parkinson’s disease (L-DOPA-induced dyskinesia in 6-OHDA-lesioned rats)	Male rats; 6-OHDA lesion; ALA (31.5 or 63 mg/kg, i.p.) co-administered with L-DOPA over the treatment period (chronic administration model)	↓ Abnormal involuntary movements (dyskinesia); ↓ oxidative stress (↓ MDA, ↑ GSH); ↓ neuroinflammation and apoptosis in substantia nigra	[81]
Nanomicellar complex of carnosine and ALA (CLA)—Rotenone-induced Parkinson’s disease model	Male rats; rotenone-induced PD model; nanomicellar CLA administered intraperitoneally (25 or 50 mg/kg) daily for 18 days	↓ Muscle rigidity; ↑ locomotor activity; ↑ total brain antioxidant activity; ↑ neuronal density in substantia nigra; ↑ striatal dopamine levels (neuroprotective and antioxidant effects)	[82]
Long-term relapsing-remitting EAE (Experimental Autoimmune Encephalomyelitis)	ALA (100 mg/kg/day) intraperitoneally, evaluated at 7 days and 180 days post-onset	↓ MBP; ↓ β-APP expression; down-regulated TNF-α and up-regulated TGF-β 7 days after onset; ↓ MDA; ↑ SOD; ↑ Treg levels at 7 days after onset	[85]
Focal-cortical EAE model	C57BL/6 mice with focal cortical EAE induced by intracortical TNF-α/IFN-γ injection; ALA administered subcutaneously (100 mg/kg/day), with lesions analyzed 3 days after induction	↓ Cortical CD4^+^ inflammatory infiltrates; ↓ galectin-3^+^ activated microglia/macrophages; ↓ CD45^+^ infiltrating cells	[86]
Transgenic ALS mice carrying the G93A Cu/Zn superoxide dismutase (SOD1) mutation	ALA administration: Dietary supplementation. Dose: NR (not specified in abstract). Duration: Chronic, until end-stage/survival analysis (exact duration not specified)	↑ Survival (statistically significant improvement vs. untreated G93A mice); ↓ Disease progression (inferred from survival extension); ↓ Oxidative stress; ↑ Mitochondrial/redox support; Neuroprotection consistent with antioxidant and metabolic modulation rather than GAPDH-dependent anti-apoptotic signaling	[89]
*Drosophila* models harboring mutant hSOD1 (G85R, G93A) + NSC34 Motor neuron-like cell line expressing mutant hSOD1	Transgenic *Drosophila* fed ALA-supplemented food at 2 mM concentration for 30 days post-eclosion; NSC-34 cells expressing hSOD1 mutants treated with 100 µM ALA for 24 h	↑ Climbing ability and lifespan in hSOD1-mutant flies; ↓ ROS accumulation; restored antioxidant enzyme activity; ↓ H_2_O_2_-induced cytotoxicity in NSC-34 cells; ↑ cell viability; activated ERK/Akt signaling pathway	[90]
Transgenic mouse models of Huntington’s disease (R6/2 and N171-82Q)	Diet 0.005% (100 mg/kg/day), began at four weeks and continued throughout life	↑ Survival in both HD mouse lines; delayed weight loss and disease progression	[94]
Rat model of Huntington’s disease induced by 3-nitropropionic acid (3-NP)	Rats received ALA (100 mg/kg i.p.) and ALCAR (100 mg/kg i.p.) daily for 28 days, starting with 3-NP exposure	Restored mitochondrial complex II and IV activity; ↑ ATP levels; ↓ ROS; ↓ MDA, protein carbonyls; ↑ SOD; ↑ catalase; ↑ learning and memory; inhibition of apoptotic pathways	[95]
Mechanistic/in vitro or non-ND models			
In vitro HDAC assays and mammalian cell models	In vitro; lipoic acid/lipoamide evaluated for HDAC inhibition and associated acetylation changes	Supports an epigenetically relevant regulatory layer beyond redox and metabolic actions	[17]
*H. pylori*-infected gastric epithelial AGS cells	ALA pretreatment (5 µM), co-treatment with *H. pylori* (strain NCTC11637) for 8 h in AGS cells	↑ Nuclear Nrf2/↑ HO-1 expression → ↓ ROS levels → ↓ IL-8 expression and secretion in H. pylori-infected cells	[16]
BV-2 microglia (LPS model)	Pretreatment with ALA (50–400 µM), LPS (1 µg/mL) for 30 min and ALA treatment for 24 h	↓ pro-inflammatory cytokines (TNF-α, IL-6); ↓ ROS and NO production; ↓ ERK/p38 phosphorylation; ↓ NF-κB and NLRP3 inflammasome activation; ↑ M2 phenotype-associated genes (MRC1, ARG1); ↓ M1 phenotype-associated genes (IL-1β, ICAM-1)	[18]
Subarachnoid hemorrhage (SAH) in SD rats	Sprague-Dawley rats; SAH induced by endovascular perforation; ALA 100 mg/kg i.p. administered 30 min before SAH and then daily for 3 days	↓ STING-NLRP3 inflammasome activation, ↓ IL-1β and IL-18, ↓ neuronal apoptosis, ↓ brain edema, ↑ neurological scores → neuroprotection via suppression of STING-mediated innate immune signaling	[19]
Human PBMCs, Jurkat T cells	In vitro study; ALA 10–100 µM (1–24 h) in activated human PBMCs and Jurkat T cells	↑ cAMP levels → activation of PKA → phosphorylation of CREB (Ser133) → ↓ TNF-α, IFN-γ, IL-2 production; anti-inflammatory effect independent of the EP2 receptor	[20]
Male CD-1 mice subjected to permanent middle cerebral artery occlusion	ALA (50 mg/kg i.p.), 30 min prior to ischemia; evaluation at 24 h post-ischemia	↓ Neurological deficit score; ↓ infarct volume; ↓ brain edema → ↑ SIRT1 expression; ↑ PGC-1α expression; ↑ SOD activity → neuroprotection via SIRT1-dependent PGC-1α up-regulation	[22]
Primary rat astrocyte cultures exposed to induced oxidative stress and iron overload	Pretreatment with ALA (50 µM) and Vitamin D_3_ (10 nM) → cells challenged with H_2_O_2_ (200 µM) + Fe^2+^ (100 µM) for 24 h	↓ ROS production; ↓ intracellular iron accumulation; ↑ glutathione (GSH); ↑ mitochondrial membrane potential; ↓ astrocyte senescence markers → protective effect via combined antioxidant and iron-homeostasis modulation	[25]
Rat Schwann cells (high-glucose exposure)	ALA (1, 10, 50, 100 µM) → 24–48 h exposure under CHG (30 mM) or IHG cycling (30 ↔ 5.5 mM glucose)	↓ ROS and oxidative stress → attenuation of mitochondrial apoptotic pathway activation (↓ Bax, ↓ cytochrome c release/AIF translocation) → ↓ caspase-3/9 activation and PARP cleavage; ↑ Bcl-2 expression → improved Schwann cell survival	[26]
NG108-15 neuronal hybrid cells	Pretreatment with R-LA (50 μM, 2 h) followed by H_2_O_2_ (400 μM, 24 h)	↑ neuroprotection via PI3K–Akt/GSK-3β activation and NF-κB/cytokine suppression (↓ ROS, ↓ apoptosis)	[27]
Diabetic peripheral neuropathy in Sprague–Dawley rats	In vivo; STZ-induced diabetes + HFD; ALA 60 mg/kg/day for 12 weeks	↑ Motor and sensory NCV; ↑ GSH; ↓ MDA; ↑ p-AMPK → ↑ Nrf2/HO-1/NQO1; ↓ FoxO3a/Bim → ↓ oxidative stress and apoptosis in DRG	[30]
Pilocarpine-induced seizures in Wistar rats	In vivo; pilocarpine 400 mg/kg i.p.; ALA 20 mg/kg i.p. 30 min before seizure; evaluated 24 h post-seizure	↓ Hippocampal cell death; inhibition of caspase-dependent (cyt c → casp-3) and independent (AIF translocation) pathways → neuroprotection against epileptic damage	[63]
Traumatic brain injury (TBI) in Sprague—Dawley rats	In vivo; cortical impact TBI model; ALA 100 mg/kg i.p., once daily for 3 days post-injury	↓ Neuronal apoptosis; ↓ cytochrome c release; ↓ caspase-9/-3 activation; ↑ Bcl-2/Bax ratio; preservation of mitochondrial membrane potential → neuroprotection via inhibition of mitochondrial apoptotic pathway	[64]
SH-SY5Y-APP695 cells and SH-SY5Y-MOCK (cellular Alzheimer’s model vs. control)	In vitro; ALA at 100 µM and 1 mM for 24 h (and 1 h pre-treatment for rotenone challenge) in APP695 and MOCK cells	↑ ATP; ↑ MMP in control cells; ↓ ROS in both cell types; → enhanced mitochondrial function, modest protection against rotenone-induced stress	[69]
BV-2 mouse microglial cells (Aβ1–42 oligomers exposure)	In vitro; BV-2 cells pretreated with ALA (10–100 µM, 2 h) then exposed to Aβ1–42 oligomers (2 µM, 24 h)	↑ Phagocytosis of Aβ1–42; ↓ NF-κB, NLRP3, p-p38 MAPK, and pro-inflammatory cytokines (IL-1β, TNF-α, IL-6); activation of PPAR-γ and PI3K/Akt → shift toward a neuroprotective microglial phenotype	[73]
Biotin (Vitamin B7)			
Preclinical in vivo studies (Neurodegenerative models)			
*Drosophila melanogaster* expressing human mutant tau (R406W)	In vivo; dietary biotin 10 µM from L1 larval stage to 10-day-old adults (~15–18 days total)	Restored mitochondrial membrane potential and morphology; ↑ ATP, ↓ ROS, ↓ neuronal degeneration; improved locomotor performance → neuroprotection via enhanced mitochondrial metabolism and biotin-dependent carboxylase activity	[49]
Rat model of Alzheimer’s disease (Aβ injection into lateral ventricle)	In vivo; oral biotin 10 mg/kg/day × 28 days (+/− swimming training) before and after Aβ injection	↑ Learning and memory; ↓ anxiety and depression-like behavior; ↓ MDA; ↑ total thiols; ↓ Aβ plaques and neuronal loss (CA1) → antioxidant and neuroprotective effects	[75]
*Drosophila melanogaster* model of manganese (Mn) toxicity-human derived midbrain dopaminergic neurons	Biotin supplementation in diet of flies; human derived dopaminergic neurons cultured with biotin and Mn exposure	↑ Locomotor/climbing behavior in flies; preserved dopaminergic neurons (TH^+^); mitigated mitochondrial dysfunction and neuronal loss	[83]
Dysmyelinating shiverer mice (murine oligodendrocytes) + human oligodendrocyte progenitor cell grafts	Shiverer (Shi/Shi:Rag2-/-) mice treated via maternal MD1003 in diet (5 mg/kg/day) starting P0; pups exposed in utero + via milk; grafted with human NPC-derived OPCs at P1; analyses at 12 and 20 weeks post treatment/graft	↑ Number and differentiation potential of endogenous murine oligodendroglia (↑ OLIG2^+^/CC1^+^) and of grafted human oligodendroglia; without significantly increasing axonal myelination	[88]
Biotin (Mg-biotin complex)/LPC-induced hippocampal demyelination in rats	In vivo; rats given biotin at 0.9 mg/rat/day (B1) or 9 mg/rat/day (B2) and MgB at 0.9 mg/rat/day (MgB1) or 9 mg/rat/day (MgB2); duration post-LPC demyelination (remyelination phase)	↑ Remyelination; ↑ spatial memory; ↓ NF-κB p65; ↑ BDNF, GAP43, ICAM -1; ↓ GFAP gliosis → dose-dependent beneficial effect of biotin/MgB on remyelination and neuronal transmission	[89]
Huntington’s disease mouse model (R6/1 HD mice) with OL and OPC maturation deficits	Daily combined thiamine 50 mg/kg + biotin 20 mg/kg, i.p. started at 8 weeks of age, continued for 7 weeks, euthanized/analyzed at 15 weeks	Rescue of OL/OPC maturation state; normalization of dysregulated maturation signatures and OL-lineage DEGs; ↓ neuronal pathology	[97]
Mechanistic/in vitro, ex vivo or non-ND models			
Biotin (maternal supplementation)/Female rat offspring (post-weaning fructose)	Maternal global caloric restriction during gestation + biotin 2 mg/kg for dams; offspring exposed to 20% fructose in drinking water for 16 weeks after weaning	↓ cardiometabolic risk markers, including hypertriglyceridemia, hypercholesterolemia, hepatic steatosis, glucose intolerance, insulin resistance, hypertension, and vascular hyperresponsiveness	[37]
Pancreatic rat islets	In vitro/ex vivo; isolated rat islets treated with biotin (50 µM) for 2–24 h; measurement of mRNA, ATP content	↑ Glucokinase mRNA; ↑ ATP content; effect blocked by sGC or PKG inhibitors → Mechanism: sGC/PKG signaling → ↑ ATP → autocrine insulin release → PI3K/Akt activation	[38]
Wistar rat aortic rings	Ex vivo; isolated rat aortic rings; biotin 10 nM incubation with or without antihypertensive drugs (BMY 7378 100 nM; captopril 1 µM; nitrendipine 100 nM) during vasoconstriction assays	↑ Relaxation/enhanced vasodilatory response with antihypertensive drugs; ↓ phenylephrine- and Ca^2+^-induced contraction → vascular protective/vasorelaxant effect of biotin	[39]
Murine oligodendrocytes (158N)	Pre- and co-treatment with biotin (10–100 μM) for 24–48 h before/with 7β-OHC challenge	↑ Cell survival via attenuation of oxidative stress, preservation of mitochondrial function (ΔΨm ↑, ATP ↑), and normalization of lipid metabolism under 7β-OHC-induced toxicity	[44]
Male BALB/cAnN Hsd mice	Dietary supplementation: control diet 1.76 mg free biotin/kg diet vs. biotin-supplemented diet 97.7 mg free biotin/kg diet; 8 weeks post-weaning	↓ Serum triglycerides; ↓ hepatic triglycerides; ↑ Hepatic cGMP; ↑ p-AMPK and ↑ p-ACC-1; ↓ Mature SREBP-1c and ↓ FAS → ↓ lipogenesis	[45]
Murine adipocyte cell line (3T3-L1)	Control media vs. biotin-supplemented media (1 μM biotin); 8 days (main experimental condition; dose–response evaluated but downstream assays performed at 1 μM)	↑ p-AMPK (T172); ↑ p-ACC-1 (S79) and ↑ p-ACC-2 (S212); ↓ Fatty acid synthesis; ↑ Fatty acid uptake; FA transport/activation genes: ↑ Fatp1 mRNA, ↑ Acsl1 mRNA; ↑ GPAT-3 protein; Triglyceride content ↔; ↑ Lipid droplet number with droplet area/size	[46]
Abcd1^−^ mouse model of X-ALD	In vivo; high-dose pharmaceutical-grade biotin (MD1003) in Abcd1 knockout mice; treated for months (starting at ~13 months old mice, followed until ~18 months)	↑ NRF2-driven antioxidant response; ↑ mitochondrial biogenesis (PGC-1α) and ATP; ↓ axonal degeneration and locomotor impairment; normalized SREBP-1c/mTORC1 lipid-metabolism program → restored redox, energy and lipid homeostasis	[47]
Post-natal rat-derived oligodendrocyte lineage cells	OPCs (A2B5^+^) cultured; biotin (2.5 to 250 µg/mL), ensheathment assessed on nanofiber assay (3 days)	↓ Cell death under glucose-free conditions; ↑myelin-like ensheathment; ↑ % ensheathing cells, number and length of segments; ↑ ATP production	[49]
Human PBMCs obtained from healthy adults	In vivo supplementation, pre–post design: biotin 3.1 μmol/day (≈0.75 mg/day) for 14 days; blood/urine collected pre- vs. post-supplementation; PBMC stimulated ex vivo with concanavalin A (up to 3 days) to mimic antigenic activation	↓ PBMC proliferation; IL-1β secretion ↓; IL-2 secretion ↓; PBMC subset proportions (CD markers) ↔ no change after 14 days	[51]
Human PBMC (stimulated ex vivo with concanavalin A (ConA) to mimic antigenic activation	In vivo supplementation (pre–post): biotin 8.8 μmol/day for 21 days; PBMC isolated before vs. after supplementation; ex vivo ConA stimulation 21 h	After biotin vs. before biotin; ↑ fold-changes: IFN-γ mRNA (~4.3×); ↑ IL-1β mRNA (~5.6×); ↑ 3-Methylcrotonyl-CoA carboxylase mRNA (~8.9×); ↓ IL-4 mRNA (reported as ~6.8× higher before supplementation) → reduced after	[52]
Male Wistar rats with propionic acid (PPA)-induced autism-like features	PPA: 500 mg/kg/day, s.c., 5 days (autism-like induction); MgB (oral gavage): 160.7 µg/day (MgBI), 1606.9 µg/day (MgBII), 8034.4 µg/day (MgBIII), 2 weeks (doses derived from human-equivalent 10/100/500 mg biotin)	↑ Social behavior and anxiety-like metrics; ↑ learning/memory; ↓ MDA (serum/brain); ↑ CAT; ↑ SOD; ↑ GPx; ↑ GSH; ↓ TNF-α; ↓ IL-6; ↓ IL-17; ↓ CCL-3, ↓ CCL-5; ↓ CXCL-16; ↑ counter-regulators OPG, ↑ MMP-9; Neurotransmission/trophic markers improved: ↑ serotonin, ↑ dopamine; ↑ BDNF, ↑ GAP-43, ↑ ICAM-1, ↑ PSD-93, ↑ PSD-95; ↓ GFAP; ↑ Purkinje cell number/size/density; ↓ hippocampal disorganization; ↑ Carboxylase-related “neurodevelopment markers”: ↑ ACC-1, ↑ ACC-2, ↑ PC, ↑ PCC, ↑ MCC	[53]
Murine models of intestinal inflammation	Oral biotin supplementation (≈1–10 mg/kg/day; duration 7–21 days, depending on model)	↓ NF-κB activation and downstream pro-inflammatory cytokines (TNF-α, IL-1β, IL-6); ↑ intestinal barrier integrity and epithelial tight-junction preservation → anti-inflammatory	[54]
Dextran sulfate sodium (DSS)-induced colitis in mice	In vivo; DSS-induced colitis model in mice with oral biotin supplementation (≈1–5 mg/kg/day) administered throughout DSS exposure (5–7 days) and recovery phase (up to 14 days)	↓ NF-κB activation; ↓ pro-inflammatory cytokine expression (TNF-α, IL-1β, IL-6); ↑ intestinal barrier integrity; ↓ disease activity index → anti-inflammatory and barrier-protective	[56]
High-fructose diet-induced metabolic syndrome in Wistar rats	In vivo; male Wistar rats fed 30% fructose in drinking water for 12 weeks, then biotin i.p. 2 mg/kg/day for 4 weeks	↓ Plasma TG, cholesterol, LDL-c, transaminases, blood pressure; ↑ HDL-c; improved glucose and insulin tolerance; ↓ hepatic steatosis and oxidative stress	[61]

Abbreviations: ↑, increase; ↓, decrease; ↔, no significant change; Aβ, amyloid-β; Aβ1–42, amyloid-beta 1–42 peptide; 6-OHDA, 6-hydroxydopamine; A2B5, oligodendrocyte progenitor cell surface marker; ACC-1, acetyl-CoA carboxylase 1; ACC-2, acetyl-CoA carboxylase 2; Acsl1, acyl-CoA synthetase long-chain family member 1; AIF, apoptosis-inducing factor; AGS, human gastric adenocarcinoma cell line; Akt, protein kinase B; ALA, α-lipoic acid; ALCAR, acetyl-L-carnitine; ALS, amyotrophic lateral sclerosis; AMPK, AMP-activated protein kinase; APP, amyloid precursor protein; ARG1, arginase 1; Asp, aspartate; ATP, adenosine triphosphate; B1/B2, low- and high-dose biotin groups; Bax, Bcl-2-associated X protein; BDNF, brain-derived neurotrophic factor; Bcl-2, B-cell lymphoma-2; BNIP3L, BCL2-interacting protein 3-like; BV-2, murine microglial cell line; CAT, catalase; CLA, nanomicellar complex of carnosine and α-lipoic acid; CCL3, C-C motif chemokine ligand 3; CCL5, C-C motif chemokine ligand 5; CHG, constant high glucose; ConA, concanavalin A; CREB, cAMP response element-binding protein; cyt c, cytochrome c; CXCL16, C-X-C motif chemokine ligand 16; DDS, dapsone (diaminodiphenyl sulfone); DEGs, differentially expressed genes; DMT1, divalent metal transporter 1; DRG, dorsal root ganglion; DSS, dextran sulfate sodium; EAE, experimental autoimmune encephalomyelitis; EP2, prostaglandin E2 receptor subtype 2; ERK, extracellular signal-regulated kinase; EX527, selective SIRT1 inhibitor; FA, fatty acids; FAS, fatty acid synthase; Fatp1, fatty acid transport protein 1; Fe^2+^, ferrous iron; FPN, ferroportin; FTH1, ferritin heavy chain 1; FoxO3a, forkhead box O3a; GAP-43, growth-associated protein 43; GFAP, glial fibrillary acidic protein; GLUT3, glucose transporter 3; GLUT4, glucose transporter 4; Gln, glutamine; Glu, glutamate; GPAT-3, glycerol-3-phosphate acyltransferase 3; GPx, glutathione peroxidase; GPX4, glutathione peroxidase 4; GSH, reduced glutathione; GSK-3β, glycogen synthase kinase-3 beta; H_2_O_2_, hydrogen peroxide; HD, Huntington’s disease; HDAC, histone deacetylase; HDL-c, high-density lipoprotein cholesterol; HFD, high-fat diet; HIF-1α, hypoxia-inducible factor-1 alpha; HK, hexokinase; HO-1, heme oxygenase-1; ICAM-1, intercellular adhesion molecule 1; Iba-1, ionized calcium-binding adaptor molecule 1; IFN-γ, interferon gamma; IHG, intermittent high glucose; IL, interleukin; IRP2, iron-regulatory protein 2; JNK, c-Jun N-terminal kinase; LDL-c, low-density lipoprotein cholesterol; LPC, lysophosphatidylcholine; LPS, lipopolysaccharide; MBP, myelin basic protein; MCC, 3-methylcrotonyl-CoA carboxylase; MDA, malondialdehyde; MD1003, pharmaceutical-grade high-dose biotin formulation; MgB, magnesium biotinate; MgBI/MgBII/MgBIII, low-, medium- and high-dose magnesium biotinate groups; MMP, mitochondrial membrane potential; MMP-9, matrix metalloproteinase-9; MPP^+^, 1-methyl-4-phenylpyridinium; MPTP, 1-methyl-4-phenyl-1,2,3,6-tetrahydropyridine; MRC1, mannose receptor C-type 1; MS, multiple sclerosis; MWM, Morris water maze; µPET, micro-positron emission tomography; NCV, nerve conduction velocity; NF-κB, nuclear factor kappa-B; NK cells, natural killer cells; NLRP3, NLR family pyrin domain containing 3; NQO1, NAD(P)H quinone oxidoreductase 1; NRF2, nuclear factor erythroid 2-related factor 2; NSC-34, motor neuron-like cell line; NPC, neural progenitor cells; OL, oligodendrocyte; OLIG2, oligodendrocyte lineage transcription factor 2; OPC, oligodendrocyte progenitor cell; OPG, osteoprotegerin; PARP, poly(ADP-ribose) polymerase; PBMCs, peripheral blood mononuclear cells; PC, pyruvate carboxylase; PC12, rat pheochromocytoma cell line; PFC, prefrontal cortex; PGC-1α, peroxisome proliferator-activated receptor gamma coactivator-1 alpha; PI3K, phosphoinositide 3-kinase; PKA, protein kinase A; PKG, protein kinase G; PPAR-γ, peroxisome proliferator-activated receptor gamma; PPA, propionic acid; PSD-93, postsynaptic density protein 93; PSD-95, postsynaptic density protein 95; R-LA, R-alpha-lipoic acid; ROS, reactive oxygen species; SAH, subarachnoid hemorrhage; SH-SY5Y, human neuroblastoma cell line; SIRT1, sirtuin-1; SLC7A11 (xCT), cystine/glutamate antiporter; SN, substantia nigra; SOD, superoxide dismutase; STING, stimulator of interferon genes; STZ, streptozotocin; TBARS, thiobarbituric acid-reactive substances; TCA cycle, tricarboxylic acid cycle; TEAC, Trolox equivalent antioxidant capacity; TG, triglycerides; TH, tyrosine hydroxylase; TGF-β, transforming growth factor beta; TNF-α, tumor necrosis factor alpha; Tregs, CD4^+^CD25^+^FoxP3^+^ regulatory T cells; X-ALD, X-linked adrenoleukodystrophy; ΔΨm, mitochondrial membrane potential.

**Table 2:** Clinical Studies of Alpha-Lipoic Acid and Biotin in Neurodegenerative Diseases.

Type/Population/Model	Design/Dose/Duration	Primary Outcome	Comment (Quality)	Ref.
Alpha-Lipoic Acid (ALA)—Clinical studies				
Alzheimer’s disease (probable AD), age ≥ 55; MMSE 15–26	Randomized, double-blind, placebo-controlled, 3-arm pilot (*n* = 39; 13/group); ω-3 fish oil 3 g/day (DHA 675 mg/day + EPA 975 mg/day) ± alpha-lipoic acid 600 mg/day; 12 months	Primary: urinary F2-isoprostanes ↔ (no between-group difference at 12 months). Secondary: ↓ MMSE decline in ω-3 + ALA vs. placebo; ↓ IADL decline in ω-3 and ω-3 + ALA vs. placebo; ↔ ADAS-Cog; ADL	Moderate internal validity (randomized, double-blind, placebo-controlled; 12-month follow-up; prespecified primary endpoint). Limitations: small pilot sample (underpowered for clinical endpoints); baseline imbalance in F2-isoprostanes; no ALA-only arm, so synergy vs. ALA effect alone cannot be isolated.	[99]
Prospective observational study/Alzheimer’s disease (mild–moderate; MMSE 12–26), stratified by type 2 diabetes presence vs. absence	ALA 600 mg/day (oral) given adjunctively (with standard antidementia care), comparing outcomes in AD + DM (Group A) vs. AD without DM (Group B); follow-up up to 16 months	↑ Cognition/function within groups, with greater improvement in AD + DM: ↑ proportion with MMSE improvement (43% in AD + DM vs. 23% without DM); ↓ ADAS-Cog (improved), ↑ CIBIC(+)(improved), ↑ ADFACS (improved) effects reported as greater in AD + DM. ↓ Insulin resistance over follow-up, proposed as a contributor	Low–moderate evidence strength: not randomized, no placebo arm, group comparison is by DM status (not treatment assignment), substantial confounding risk. The authors explicitly note that the study is observational and cannot establish definitive efficacy	[100]
Randomized, double-blind, placebo-controlled/Mild–moderate Alzheimer’s disease	3-arm randomization for 16 weeks: Vitamin E 800 IU/day + Vitamin C 500 mg/day + α-lipoic acid 900 mg/day (E/C/ALA) vs. CoQ10 400 mg TID vs. placebo	CSF F2-isoprostanes ↓ (~19%) in the E/C/ALA arm; CSF Aβ42 ↔, tau ↔, p-tau181 ↔. Cognition: MMSE decline ↑ (faster decline) in the E/C/ALA arm	Strong internal validity (randomized, double-blind, placebo-controlled; biomarker-focused). Limitations: short duration (16 weeks); biomarker endpoints may not translate to clinical benefit; signal of faster cognitive decline in E/C/ALA warrants caution	[102]
Clinical trial, Phase 1/2, open-label/Probable progressive supranuclear palsy (PSP; atypical parkinsonism), single-site small cohort	Open-label daily supplementation with ALA 600 mg/day combined with L-acetyl-carnitine 1.5 g/day for approximately 6 months in a small exploratory cohort (*n* ≈ 11)	Primary: safety/tolerability. Common adverse events included restlessness, dizziness, insomnia, and seizures. Exploratory efficacy and biomarker outcomes were listed but were not adequately reported or remain unpublished	Very low evidence strength due to exploratory design, very small sample (~11 participants), lack of randomization and placebo/control group, and absence of peer-reviewed outcome data. Informative mainly as a feasibility and safety signal rather than evidence of clinical efficacy	[107]
Clinical trial, randomized placebo-controlled pilot/MS subjects (*n* = 37)	Randomized to placebo or oral ALA: 600 mg BID, 1200 mg QD, or 1200 mg BID for 14 days	↓ Biomarkers of immune cell migration: ↓ serum MMP-9 (higher peak ALA exposure associated with greater MMP-9 reduction) and ↓ sICAM-1 with a dose–response relationship; tolerability generally acceptable	Strengths: randomized placebo arm; clear dosing arms; objective PK + biomarker endpoints. Limitations: very short duration (14 days); not powered for clinical disability outcomes; biomarker changes are surrogate (clinical benefit not established).	[110]
Clinical trial, randomized, double-blind, placebo-controlled pilot/SPMS participants	Oral alpha-lipoic acid 1200 mg/day vs. placebo; 2 years	↑ Walking performance (modest): ↓ Timed Up and Go (TUG) time (medium effect at 2 years); in lower-disability subgroup (EDSS < 6, no assistive device), ↓ turning time (significant). ↔ no between-group differences in balance metrics	Solid design (double-blind RCT) with objective sensor-based outcomes, but pilot sample size and small cohort limit precision; subgroup finding is hypothesis-generating; clinical disability endpoints not primary	[111]
Mechanistic pharmacokinetic study/Healthy controls, RRMS, and SPMS	Single-dose, within-subject (pre–post) design: after standardized breakfast + baseline draw, participants ingested racemic ALA 1200 mg PO (4 × 300 mg capsules); blood collected at 0, 1, 2, 3, 4 h (acute exposure)	↑ PBMC cAMP at 2 and 4 h in healthy controls and SPMS; ↓ PBMC cAMP in RRMS (divergent response). PK: ↔ plasma ALA (no significant between-group PK differences). PGE2: ↓ in female RRMS, plasma PGE2 at 4 h vs. female HC/SPMS	Strong for mechanism-of-action inference (objective PK + cellular second messenger readout), but limited clinical inference: no placebo/control, acute single-dose window, and endpoints are surrogate biomarkers rather than disability/relapse outcomes.	[111]
Clinical trial, randomized parallel-group/ALS patients (planned *n* = 150; China; age 20–75 years; revised El Escorial criteria; disease onset < 2 years; baseline FVC ≥ 70%)	Randomized to lipoic acid vs. control; 6 treatment courses (~5 months), with assessments at baseline, course 3, and course 6. Dose: NR in accessible registry summaries	Planned outcomes included motor function and disease progression (ALSFRS-R, ROADS, UMN scale, muscle strength scale, EMG) and respiratory function (including FVC); planned safety outcomes included routine blood and urine testing, liver and kidney function, and coagulation indices	Protocol-level evidence only. It is appropriate for describing registered clinical testing, but no conclusions regarding efficacy or safety can be drawn until results are posted or published. Randomization supports internal validity in principle, although risk of reporting bias remains in the absence of available outcome data	[119]
Pilot RCT/SPMS adults (single-site; total randomized *n* = 51)	Double-blind, randomized, placebo-controlled; alpha-lipoic acid (ALA) 1200 mg/day orally vs. placebo for 2 years	Brain atrophy ↓: annualized percent change brain volume (PCBV) significantly less negative with ALA (reported as ~68% reduction vs. placebo); exploratory clinical outcomes suggested possible benefit, but primary signal is MRI atrophy. Safety: serious renal events ↑ (reported in ALA arm)	Good internal validity for a pilot study (randomized, double-blind; Class I evidence for primary MRI endpoint). Limits: small sample/single center; clinical endpoints underpowered; safety signal (renal events) warrants caution.	[124]
Biotin—Clinical studies				
Clinical trial, phase 3 (pivotal) randomized, double-blind, placebo-controlled trial/SPMS and PPMS	MD1003 (biotin 100 mg) orally TID (300 mg/day) vs. placebo for 12 months, then extension phase with MD1003 for all patients (trial included 154 randomized)	↑ Disability reversal at month 9 confirmed at month 12: 12.6% MD1003 vs. 0% placebo (*p* = 0.005). Disability reversal defined as EDSS ↓ ≥1.0 (or ≥0.5 if EDSS 6–7) or T25FW ↓ ≥20% vs. best baseline. Secondary: ↓ EDSS progression and ↑ clinician global impression improved vs. placebo	Strong design (double-blind RCT; prespecified composite primary endpoint). Limitations: modest responder proportion; endpoint combines EDSS/T25FW (functional heterogeneity). Safety reported as similar to placebo over the blinded phase. Potential COI reported	[35]
Prospective cohort/UK Biobank adults (40–69 years at baseline); outcome: incident all-cause dementia and Alzheimer’s disease	Exposure: dietary biotin intake estimated via 24 h recall and categorized into quartiles (Q1–Q4). Follow-up: median 11.25 years; 1256 incident dementia cases. (No intervention dose; this is intake, not supplementation	↓ Dementia risk with higher dietary biotin: vs. Q1, ↓ all-cause dementia HR (Q2 0.75, Q3 0.68, Q4 0.67). ↓ AD risk ↓ (Q2 0.74; Q3 0.65; nonlinear association reported). Inflammation mediation: SII partially mediates the association	Large sample, prospective design, extensive covariate adjustment + sensitivity analyses. Key limitations: observational residual confounding, dietary recall measurement error, and dementia defined via algorithmic linkage (not uniform clinical adjudication). Cannot infer causality or supplementation efficacy	[103]
Clinical, pilot proof-of-concept; uncontrolled, non-blinded/Progressive MS (PPMS + SPMS); *n* = 23	High-dose biotin 100–300 mg/day (oral); treatment 2–36 months (mean ~9 months)	Visual function improved in a subset of patients with optic-nerve involvement (↑ visual acuity; VEP P100 responses improved or reappeared in some cases). Exploratory metabolic imaging findings were also reported	Low evidence strength; uncontrolled observational series. Findings derive from uncontrolled clinical experience rather than from a randomized trial and should be interpreted cautiously	[113]
Retrospective real-world case series/Progressive MS (*n* ≈ 43)	Pharmaceutical-grade biotin 300 mg/day (oral); follow-up up to ~1 year	No sustained disability improvement; EDSS unchanged overall and clinical worsening observed in ~38–43% of patients, with some individuals improving after treatment discontinuation. Safety laboratory parameters remained largely stable	Retrospective real-world clinical experience without randomization or placebo control; results reflect observational outcomes rather than controlled efficacy evidence	[113]
Randomized, double-blind, placebo-controlled (RCT)/MS patients with chronic visual loss after optic neuritis: either AON (acute optic neuritis) or PON (progressive optic neuropathy)	Randomized 2:1 to MD1003 (biotin 300 mg/day, oral; 100 mg TID) vs. placebo for 6 months, then 6-month open-label extension	Primary (month 6): visual acuity at 100% contrast (logMAR) ↔ overall. Subgroup: PON showed a trend toward benefit (↑ 100% contrast visual acuity), with ↑ RNFL thickness ↔/trend and ↑ health outcomes ↔/trend; ↔ AON	Strong internal validity (double-blind RCT; prespecified primary endpoint). Limitations: primary endpoint negative; subgroup findings are exploratory; sensitivity of visual outcomes and heterogeneity (AON vs. PON) may dilute effects. Funding by MedDay Pharmaceuticals (potential COI).	[114]
Phase 3 RCT/Progressive MS (SPMS and PPMS); multicenter (90 sites, 13 countries); randomized MD1003 *n* = 326 vs. placebo *n* = 316	Randomized, double-blind, parallel-group, placebo-controlled. MD1003 (biotin) 100 mg TID = 300 mg/day (oral) vs. placebo. Primary endpoint assessed at month 12 with confirmation at month 15. Mean time in placebo-controlled phase 20.1 months (range 15–27)	Disability improvement ↔ (not significant): improved at month 12 (confirmed at month 15) in 12% (39/326) MD1003 vs. 9% (29/316) placebo; OR 1.35 (95% CI 0.81–2.26). No significant benefit on disability or walking outcomes overall	High-quality design (large Phase 3 double-blind RCT). However, primary endpoint negative; conclusion is that MD1003 cannot be recommended for progressive MS. Also highlights risk of harmful consequences from laboratory-test interference with high-dose biotin. Funded by MedDay Pharmaceuticals (potential conflict of interest)	[115]
Prospective cohort (observational)/UK Biobank participants (*n* = 122,959)	Dietary biotin intake estimated from 24 h dietary recall, analyzed by intake categories (quartiles). Follow-up: long-term (median ~11 years). No supplementation dose (this is intake, not an intervention)	Higher dietary biotin intake associated with ↓ dementia risk and ↓ AD risk (dose–response/nonlinear patterns reported)	Strong for association (large sample, prospective design), but cannot infer causality (residual confounding, dietary measurement error). Not evidence for therapeutic high-dose biotin efficacy	[116]
Clinical trial, Phase 2b open-label, uncontrolled pilot/15 patients with chronic demyelinating peripheral neuropathy: CIDP (*n* = 5), anti-MAG neuropathy (*n* = 5), CMT1A/1B (*n* = 5)	High-dose pharmaceutical-grade biotin 100 mg PO TID = 300 mg/day, up to 52 weeks	Primary endpoint: predefined electrophysiology improvement ↔ (primary endpoint not met): required ≥10% relative improvement in 2/4 variables (MNCV ↑, DML ↓, F-wave latency ↓, CMAP duration ↓). Secondary/exploratory: several sensory/motor parameters ↑, gait measures ↑, nerve excitability measures ↑; tolerability acceptable with AE present but serious AE not judged related.	Low–moderate evidence for efficacy: open-label, no control group, very small sample and heterogeneous etiologies → high risk of bias/confounding; best viewed as proof-of-concept/signal-generating	[117]
Clinical trial, pilot RCT/Adults with probable or definite ALS, age 25–80 (*n* = 30; MD1003 *n* = 20, placebo *n* = 10)	Randomized (2:1), double-blind, placebo-controlled; MD1003 (pharmaceutical-grade biotin) 300 mg/day PO vs. placebo for 24 weeks (6 months)	Primary (safety/tolerability): AE ↔ vs. placebo (both 60%); deaths: MD1003 2 vs. placebo 1. Efficacy (exploratory): ALSFRS-R change at month 6 ↔ (no significant difference; *p* = 0.49)	Small pilot with baseline imbalance (MD1003 group had faster pre-screening ALSFRS-R decline and more upper-limb onset), limiting efficacy inference; supports safety signal, efficacy not established → larger trials warranted.	[119]
Clinical trial, Phase 2/ALS patients	Randomized, double-blind, 2:1 allocation; biotin 300 mg/day PO vs. placebo; 6 months, visits at baseline, 3, and 6 months	Primary: adverse effects (safety/tolerability) NR. Secondary: motor disability by ALS-FRS, pulmonary function (FEV1, FVC), body weight NR	Protocol-level evidence only (no results → no conclusions on benefit). Trial design is stronger than observational studies (randomized, blinded), but risk of non-publication/reporting bias remains until results are posted.	[120]
Clinical trial, interventional/Huntington’s disease (mild–moderate stages)	Combined oral thiamine + biotin; dose escalation with lab monitoring during escalation; follow-up ~12 months. Exact mg doses: NR in the accessible registry summaries.	Primary: safety/tolerability, NR (no posted results). Key biomarker endpoint: CSF thiamine monophosphate (TMP) ↑ (planned) as the main CNS biological marker. Additional planned exploratory outcomes include progression-related biomarkers/neuroimaging (e.g., caudate/WM/cortical measures) and CSF neurofilaments (planned)	Protocol-level evidence only (no outcomes yet → no conclusions). Strength: prospective interventional design with CNS biomarker focus. Limitation: until results are available, efficacy/safety remain undetermined and susceptible to reporting bias	[122]

Abbreviations: ↑, increase; ↓, decrease; ↔, no significant change; AD, Alzheimer’s disease; ADAS-Cog, Alzheimer’s Disease Assessment Scale–Cognitive Subscale; ADL, activities of daily living; ADFACS, Alzheimer’s Disease Functional Assessment and Change Scale; AE, adverse events; ALA, α-lipoic acid; ALS, amyotrophic lateral sclerosis; ALSFRS-R, Amyotrophic Lateral Sclerosis Functional Rating Scale–Revised; AON, acute optic neuritis; BID, twice daily; CIBIC(+), Clinician’s Interview-Based Impression of Change Plus Caregiver Input; CIDP, chronic inflammatory demyelinating polyneuropathy; CMAP, compound muscle action potential; CNS, central nervous system; CoQ10, coenzyme Q10; CSF, cerebrospinal fluid; DHA, docosahexaenoic acid; DML, distal motor latency; DM, diabetes mellitus; EDSS, Expanded Disability Status Scale; EMG, electromyography; EPA, eicosapentaenoic acid; FEV1, forced expiratory volume in 1 s; FVC, forced vital capacity; HC, healthy controls; HR, hazard ratio; IADL, instrumental activities of daily living; logMAR, logarithm of the minimum angle of resolution; MMP-9, matrix metalloproteinase-9; MMSE, Mini-Mental State Examination; MRI, magnetic resonance imaging; MS, multiple sclerosis; NR, not reported; OR, odds ratio; PCBV, percent brain volume change; PGE2, prostaglandin E2; PK, pharmacokinetics; PO, per os (oral administration); PON, progressive optic neuropathy; PPMS, primary progressive multiple sclerosis; PSP, progressive supranuclear palsy; QD, once daily; RNFL, retinal nerve fiber layer; ROADS, Rasch-Built Overall Amyotrophic Lateral Sclerosis Disability Scale; RRMS, relapsing-remitting multiple sclerosis; sICAM-1, soluble intercellular adhesion molecule-1; SPMS, secondary progressive multiple sclerosis; SII, systemic immune-inflammation index; T25FW, Timed 25-Foot Walk; TID, three times daily; TUG, Timed Up and Go; UMN, upper motor neuron; VEP, visual evoked potentials.

## Data Availability

No new data were created or analyzed in this study.

## References

[1] Gadhave D.G., Sugandhi V.V., Jha S.K., Nangare S.N., Gupta G., Singh S.K., Dua K., Cho H., Hansbro P.M., Paudel K.R. (2024). Neurodegenerative Disorders: Mechanisms of Degeneration and Therapeutic Approaches with Their Clinical Relevance. Ageing Res. Rev..

[2] Lamptey R.N.L., Chaulagain B., Trivedi R., Gothwal A., Layek B., Singh J. (2022). A Review of the Common Neurodegenerative Disorders: Current Therapeutic Approaches and the Potential Role of Nanotherapeutics. Int. J. Mol. Sci..

[3] Kelser B.M., Teichner E.M., Subtirelu R.C., Hoss K.N. (2024). A Review of Proposed Mechanisms for Neurodegenerative Disease. Front. Aging Neurosci..

[4] Wilson D.M., Cookson M.R., Van Den Bosch L., Zetterberg H., Holtzman D.M., Dewachter I. (2023). Hallmarks of Neurodegenerative Diseases. Cell.

[5] Zhang Y., Li X.W., Zhang Y., Li X. (2025). Advances in Research on Mitochondrial Dysfunction in Neurodegenerative Diseases. J. Neurol..

[6] Ristori S., Bertoni G., Bientinesi E., Monti D. (2025). The Role of Nutraceuticals and Functional Foods in Mitigating Cellular Senescence and Its Related Aspects: A Key Strategy for Delaying or Preventing Aging and Neurodegenerative Disorders. Nutrients.

[7] Riveron-Negrete L., Fernández-Mejía C. (2017). Pharmacological Effects of Biotin in Animals. Mini-Rev. Med. Chem..

[8] Aguilera-Méndez A., Boone-Villa D., Nieto-Aguilar R., Villafaña-Rauda S., Saavedra-Molina A., Sobrevilla J.V. (2022). Role of Vitamins in the Metabolic Syndrome and Cardiovascular Disease. Pflug. Arch. Eur. J. Physiol..

[9] Karachaliou C.E., Livaniou E. (2024). Biotin Homeostasis and Human Disorders: Recent Findings and Perspectives. Int. J. Mol. Sci..

[10] Manavi M.A., Nourhashemi M., Emami S., Fathian Nasab M.H., Dehnavi F., Küçükkılınç T.T., Foroumadi A., Sharifzadeh M., Khoobi M. (2025). Lipoic Acid Scaffold Applications in the Design of Multitarget-Directed Ligands Against Alzheimer’s Disease. Bioorg. Chem..

[11] Superti F., Russo R. (2024). Alpha-Lipoic Acid: Biological Mechanisms and Health Benefits. Antioxidants.

[12] Wang Y., Jiang S., He Y., Pang P., Shan H. (2025). Advances in α-Lipoic Acid for Disease Prevention: Mechanisms and Therapeutic Insights. Molecules.

[13] Kodentsova V.M., Risnik D.V., Kryukova E.V., Dariy S.G. (2023). Functional Ingredients for Specialized Foods: Issues to Be Addressed. Med. Alph..

[14] Xie H., Yang X., Cao Y., Long X., Shang H., Jia Z. (2022). Role of Lipoic Acid in Multiple Sclerosis. CNS Neurosci. Ther..

[15] Molz P., Schröder N. (2017). Potential Therapeutic Effects of Lipoic Acid on Memory Deficits Related to Aging and Neurodegeneration. Front. Pharmacol..

[16] Kyung S., Lim J.W., Kim H. (2019). α-Lipoic Acid Inhibits IL-8 Expression by Activating Nrf2 Signaling in *Helicobacter pylori*-Infected Gastric Epithelial Cells. Nutrients.

[17] Wood S.H., van Dam S., Craig T., Tacutu R., O’Toole A., Merry B.J., de Magalhães J.P. (2015). Transcriptome Analysis in Calorie-Restricted Rats Implicates Epigenetic and Post-Translational Mechanisms in Neuroprotection and Aging. Genome Biol..

[18] Kim S.M., Ha J.S., Han A.R., Cho S.W., Yang S.J. (2019). Effects of α-Lipoic Acid on LPS-Induced Neuroinflammation and NLRP3 Inflammasome Activation through the Regulation of BV-2 Microglial Cell Activation. BMB Rep..

[19] Lin C., He C., Li L., Liu Y., Tang L., Ni Z., Zhang N., Lai T., Chen X., Wang X. (2024). Alpha-Lipoic Acid (ALA) Ameliorates Early Brain Injury After Subarachnoid Hemorrhage via Inhibiting STING-NLRP3 Inflammatory Signaling in SD Rats. Neuroreport.

[20] Salinthone S., Yadav V., Schillace R.V., Bourdette D.N., Carr D.W. (2010). Lipoic Acid Attenuates Inflammation via cAMP and Protein Kinase A Signaling. PLoS ONE.

[21] Gomes B.A.Q., Santos S.M.D., Gato L.D.S., Espíndola K.M.M., Silva R.K.M.D., Davis K., Navegantes-Lima K.C., Burbano R.M.R., Romao P.R.T., Coleman M.D. (2025). Alpha-Lipoic Acid Reduces Neuroinflammation and Oxidative Stress Induced by Dapsone in an Animal Model. Nutrients.

[22] Jiang T., Yin F., Yao J., Brinton R.D., Cadenas E. (2013). Lipoic Acid Restores Age-Associated Impairment of Brain Energy Metabolism through the Modulation of Akt/JNK Signaling and PGC1α Transcriptional Pathway. Aging Cell.

[23] Fu B., Zhang J., Zhang X., Zhang C., Li Y., Zhang Y., He T., Li P., Zhu X., Zhao Y. (2014). Alpha-Lipoic Acid Upregulates SIRT1-Dependent PGC-1α Expression and Protects Mouse Brain against Focal Ischemia. Neuroscience.

[24] Zhang J., Jiang Y., Dong X., Meng Z., Ji L., Kang Y., Liu M., Zhou W., Song W. (2024). Alpha-Lipoic Acid Alleviates Cognitive Deficits in Transgenic APP23/PS45 Mice through a Mitophagy-Mediated Increase in ADAM10 α-Secretase Cleavage of APP. Alzheimer’s Res. Ther..

[25] Molinari C., Morsanuto V., Ghirlanda S., Ruga S., Notte F., Gaetano L., Uberti F. (2019). Role of Combined Lipoic Acid and Vitamin D3 on Astrocytes as a Way to Prevent Brain Ageing by Induced Oxidative Stress and Iron Accumulation. Oxidative Med. Cell. Longev..

[26] Sun L.Q., Chen Y.Y., Wang X., Li X.J., Xue B., Qu L., Zhang T.T., Mu Y.M., Lu J.M. (2012). The Protective Effect of Alpha Lipoic Acid on Schwann Cells Exposed to Constant or Intermittent High Glucose. Biochem. Pharmacol..

[27] Fahmy M.I., Khalaf S.S., Elrayess R.A. (2024). The Neuroprotective Effects of Alpha Lipoic Acid in Rotenone-Induced Parkinson’s Disease in Mice via Activating PI3K/AKT Pathway and Antagonizing Related Inflammatory Cascades. Eur. J. Pharmacol..

[28] Kamarudin M.N., Mohd Raflee N.A., Hussein S.S., Lo J.Y., Supriady H., Abdul Kadir H. (2014). (R)-(+)-α-Lipoic Acid Protected NG108-15 Cells against H_2_O_2_-Induced Cell Death through PI3K-Akt/GSK-3β Pathway and Suppression of NF-κB-Cytokines. Drug Des. Dev. Ther..

[29] Capece U., Moffa S., Improta I., Di Giuseppe G., Nista E.C., Cefalo C.M.A., Cinti F., Pontecorvi A., Gasbarrini A., Giaccari A. (2022). Alpha-Lipoic Acid and Glucose Metabolism: A Comprehensive Update on Biochemical and Therapeutic Features. Nutrients.

[30] Shay K.P., Moreau R.F., Smith E.J., Smith A.R., Hagen T.M. (2009). Alpha-Lipoic Acid as a Dietary Supplement: Molecular Mechanisms and Therapeutic Potential. Biochim. Biophys. Acta.

[31] Zhang T., Zhang D., Zhang Z., Tian J., An J., Zhang W., Ben Y. (2023). Alpha-Lipoic Acid Activates AMPK to Protect against Oxidative Stress and Apoptosis in Rats with Diabetic Peripheral Neuropathy. Hormones.

[32] McCarty M.F., DiNicolantonio J.J. (2017). Neuroprotective Potential of High-Dose Biotin. Med. Hypotheses.

[33] Canda E., Kalkan Uçar S., Çoker M. (2020). Biotinidase Deficiency: Prevalence, Impact and Management Strategies. Pediatr. Health Med. Ther..

[34] Sedel F., Bernard D., Mock D.M., Tourbah A. (2016). Targeting Demyelination and Virtual Hypoxia with High-Dose Biotin as a Treatment for Progressive Multiple Sclerosis. Neuropharmacology.

[35] Tourbah A., Lebrun-Frenay C., Edan G., Clanet M., Papeix C., Vukusic S., De Sèze J., Debouverie M., Gout O., Clavelou P. (2016). MD1003 (High-Dose Biotin) for the Treatment of Progressive Multiple Sclerosis: A Randomised, Double-Blind, Placebo-Controlled Study. Mult. Scler..

[36] Wal A., Sasmal A., Singh R., Yadav P., Singh Y., Garg V., Wal P. (2023). Regulatory Role, Mechanism, and Metabolic Profile of Biotin in Gene Expression. Curr. Pharmacogenomics Pers. Med..

[37] Hassan Y.I., Zempleni J. (2006). Epigenetic Regulation of Chromatin Structure and Gene Function by Biotin. J. Nutr..

[38] Aguilera-Méndez A., Figueroa-Fierros I., Ruiz-Pérez X., Godínez-Hernández D., Saavedra-Molina A., Rios-Chavez P., Villafaña S., Boone-Villa D., Ortega-Cuellar D., Gauthereau-Torres M.Y. (2024). The Beneficial Effects of Prenatal Biotin Supplementation in a Rat Model of Intrauterine Caloric Restriction to Prevent Cardiometabolic Risk in Adult Female Offspring. Int. J. Mol. Sci..

[39] Vilches-Flores A., Tovar A.R., Marin-Hernandez A., Rojas-Ochoa A., Fernandez-Mejia C. (2010). Biotin Increases Glucokinase Expression via Soluble Guanylate Cyclase/Protein Kinase G, Adenosine Triphosphate Production and Autocrine Action of Insulin in Pancreatic Rat Islets. J. Nutr. Biochem..

[40] Aguilera-Méndez A., Espino-García R., Toledo-López Z.J., Hernández-Gallegos Z., Villafaña-Rauda S., Nieto-Aguilar R., Serrato-Ochoa D., Manuel-Jacobo G.C. (2019). Biotin Improves Relaxation of Rat Aortic Rings in Combination with Antihypertensive Drugs. PharmaNutrition.

[41] Park M.K., Yang H.W., Woo S.Y., Kim D.Y., Son D.S., Choi B.Y., Suh S.W. (2025). Modulation of Second Messenger Signaling in the Brain through PDE4 and PDE5 Inhibition: Therapeutic Implications for Neurological Disorders. Cells.

[42] Borovac J., Rai J., Valencia M., Li H., Georgiou J., Collingridge G.L., Takao K., Okamoto K. (2024). Optogenetic Elevation of Postsynaptic cGMP in the Hippocampal Dentate Gyrus Enhances LTP and Modifies Mouse Behaviors. Front. Mol. Neurosci..

[43] Tropea M.R., Gulisano W., Vacanti V., Arancio O., Puzzo D., Palmeri A. (2022). Nitric Oxide/cGMP/CREB Pathway and Amyloid-Beta Crosstalk: From Physiology to Alzheimer’s Disease. Free Radic. Biol. Med..

[44] Puzzo D., Staniszewski A., Deng S.X., Privitera L., Leznik E., Liu S., Zhang H., Feng Y., Palmeri A., Landry D.W. (2009). Phosphodiesterase 5 Inhibition Improves Synaptic Function, Memory, and Amyloid-Beta Load in an Alzheimer’s Disease Mouse Model. J. Neurosci..

[45] Sghaier R., Zarrouk A., Nury T., Badreddine I., O’Brien N., Mackrill J.J., Vejux A., Samadi M., Nasser B., Caccia C. (2019). Biotin Attenuation of Oxidative Stress, Mitochondrial Dysfunction, Lipid Metabolism Alteration and 7β-Hydroxycholesterol-Induced Cell Death in 158N Murine Oligodendrocytes. Free Radic. Res..

[46] Aguilera-Méndez A., Fernández-Mejía C. (2012). The Hypotriglyceridemic Effect of Biotin Supplementation Involves Increased Levels of cGMP and AMPK Activation. BioFactors.

[47] Moreno-Méndez E., Hernández-Vázquez A., Fernández-Mejía C. (2019). Effect of Biotin Supplementation on Fatty Acid Metabolic Pathways in 3T3-L1 Adipocytes. BioFactors.

[48] Fourcade S., Goicoechea L., Parameswaran J., Schlüter A., Launay N., Ruiz M., Seyer A., Colsch B., Calingasan N.Y., Ferrer I. (2020). High-Dose Biotin Restores Redox Balance, Energy and Lipid Homeostasis, and Axonal Health in a Model of Adrenoleukodystrophy. Brain Pathol..

[49] Lohr K.M., Frost B., Scherzer C., Feany M.B. (2020). Biotin Rescues Mitochondrial Dysfunction and Neurotoxicity in a Tauopathy Model. Proc. Natl. Acad. Sci. USA.

[50] Cui Q.L., Lin Y.H., Xu Y.K.T., Fernandes M.G.F., Rao V.T.S., Kennedy T.E., Antel J. (2020). Effects of Biotin on Survival, Ensheathment, and ATP Production by Oligodendrocyte Lineage Cells In Vitro. PLoS ONE.

[51] Shen W., Hao J., Tian C., Ren J., Yang L., Li X., Luo C., Cotma C.W., Liu J. (2008). A Combination of Nutriments Improves Mitochondrial Biogenesis and Function in Skeletal Muscle of Type 2 Diabetic Goto-Kakizaki Rats. PLoS ONE.

[52] Zempleni J., Helm R.M., Mock D.M. (2001). In Vivo Biotin Supplementation at a Pharmacologic Dose Decreases Proliferation Rates of Human Peripheral Blood Mononuclear Cells and Cytokine Release. J. Nutr..

[53] Wiedmann S., Eudy J.D., Zempleni J. (2003). Biotin Supplementation Increases Expression of Genes Encoding Interferon-Gamma, Interleukin-1Beta, and 3-Methylcrotonyl-CoA Carboxylase, and Decreases Expression of the Gene Encoding Interleukin-4 in Human Peripheral Blood Mononuclear Cells. J. Nutr..

[54] Sahin K., Orhan C., Karatoprak S., Tuzcu M., Deeh P.B.D., Ozercan I.H., Sahin N., Bozoglan M.Y., Sylla S., Ojalvo S.P. (2022). Therapeutic Effects of a Novel Form of Biotin on Propionic Acid-Induced Autistic Features in Rats. Nutrients.

[55] Helmy S.A., Samaha M.M., Abd El Salam A.S.G., Abd Elrazik N.A., El-Sayed S.M. (2025). Biotin and sulfasalazine combination therapy alleviates acetic acid-induced ulcerative colitis in rats through modulation of oxidative stress and inflammatory signaling pathways. Sci. Rep..

[56] Kuroishi T., Endo Y., Muramoto K., Sugawara S. (2008). Biotin deficiency up-regulates TNF-α production in murine macrophages. J. Leukoc. Biol..

[57] Skupsky J., Sabui S., Hwang M., Nakasaki M., Cahalan M.D., Said H.M. (2020). Biotin supplementation ameliorates murine colitis by preventing NF-κB activation. Cell. Mol. Gastroenterol. Hepatol..

[58] Kuroishi T. (2015). Regulation of immunological and inflammatory functions by biotin. Can. J. Physiol. Pharmacol..

[59] Sakurai-Yageta M., Suzuki Y. (2024). Molecular Mechanisms of Biotin in Modulating Inflammatory Diseases. Nutrients.

[60] Moretti R., Peinkhofer C. (2019). B Vitamins and Fatty Acids: What Do They Share with Small Vessel Disease-Related Dementia?. Int. J. Mol. Sci..

[61] Agrawal S., Agrawal A., Said H.M. (2016). Biotin Deficiency Enhances the Inflammatory Response of Human Dendritic Cells. Am. J. Physiol. Cell Physiol..

[62] Aguilera-Mendez A., Hernández-Equihua M.G., Rueda-Rocha A.C., Guajardo-López C., Nieto-Aguilar R., Serrato-Ochoa D., Ruíz Herrera L.F., Guzmán-Nateras J.A. (2018). Protective Effect of Supplementation with Biotin against High-Fructose-Induced Metabolic Syndrome in Rats. Nutr. Res..

[63] Shahid A., Nasir K., Bhatia M. (2025). Therapeutic Potential of Alpha-Lipoic Acid: Unraveling Its Role in Oxidative Stress and Inflammatory Conditions. Curr. Issues Mol. Biol..

[64] dos Santos P.S., Feitosa C.M., Saldanha G.B., da Rocha Tomé A., Feng D., de Freitas R.M. (2011). Lipoic Acid Inhibits Caspase-Dependent and -Independent Cell Death Pathways and Is Neuroprotective against Hippocampal Damage after Pilocarpine-Induced Seizures. Pharmacol. Biochem. Behav..

[65] Wei W., Wang H., Wu Y., Ding K., Li T., Cong Z., Xu J., Zhou M., Huang L., Ding H. (2015). Alpha Lipoic Acid Inhibits Neural Apoptosis via a Mitochondrial Pathway in Rats following Traumatic Brain Injury. Neurochem. Int..

[66] Vasudevan D., Naik M.M., Mukaddam Q.I. (2014). Efficacy and Safety of Methylcobalamin, Alpha Lipoic Acid and Pregabalin Combination versus Pregabalin Monotherapy in Improving Pain and Nerve Conduction Velocity in Type 2 Diabetes-Associated Peripheral Neuropathy: Results of a Pilot Study. Ann. Indian Acad. Neurol..

[67] Maladkar M., Tekchandani C., Dave U. (2014). Post-Marketing Surveillance of Fixed Dose Combination of Methylcobalamin, Alpha Lipoic Acid, Folic Acid, Biotin, Benfotiamine, and Vitamin B6 (Nutripathy) for the Management of Peripheral Neuropathy. J. Diabetes Mellit..

[68] Holmquist L., Stuchbury G., Berbaum K., Muscat S., Young S., Hager K., Engel J., Münch G. (2007). Lipoic Acid as a Novel Treatment for Alzheimer’s Disease and Related Dementias. Pharmacol. Ther..

[69] Seifar F., Khalili M., Khaledyan H., Amiri Moghadam S., Izadi A., Azimi A., Shakouri S.K. (2019). α-Lipoic Acid, Functional Fatty Acid, as a Novel Therapeutic Alternative for Central Nervous System Diseases: A Review. Nutr. Neurosci..

[70] Dieter F., Esselun C., Eckert G.P. (2022). Redox Active α-Lipoic Acid Differentially Improves Mitochondrial Dysfunction in a Cellular Model of Alzheimer and Its Control Cells. Int. J. Mol. Sci..

[71] Quinn J.F., Bussiere J.R., Hammond R.S., Montine T.J., Henson E., Jones R.E., Stackman R.W. (2007). Chronic Dietary Alpha-Lipoic Acid Reduces Deficits in Hippocampal Memory of Aged Tg2576 Mice. Neurobiol. Aging.

[72] Sancheti H., Kanamori K., Patil I., Díaz Brinton R., Ross B.D., Cadenas E. (2014). Reversal of Metabolic Deficits by Lipoic Acid in a Triple Transgenic Mouse Model of Alzheimer’s Disease: A 13C NMR Study. J. Cereb. Blood Flow Metab..

[73] Zhang Y.H., Yan X.Z., Xu S.F., Pang Z.Q., Li L.B., Yang Y., Fan Y.G., Wang Z., Yu X., Guo C. (2020). α-Lipoic Acid Maintains Brain Glucose Metabolism via BDNF/TrkB/HIF-1α Signaling Pathway in P301S Mice. Front. Aging Neurosci..

[74] Ko C.Y., Xu J.H., Chang Y.W., Lo Y.M., Wu J.S., Huang W.C., Shen S.C. (2022). Effects of α-Lipoic Acid on Phagocytosis of Oligomeric Beta-Amyloid1–42 in BV-2 Mouse Microglial Cells. Front. Aging Neurosci..

[75] Almasi S., Jafarzadeh Shirazi M.R., Rezvani M.R., Ramezani M., Salehi I., Pegah A., Komaki A. (2024). The Protective Effect of Biotin Supplementation and Swimming Training on Cognitive Impairment and Mental Symptoms in a Rat Model of Alzheimer’s Disease: A Behavioral, Biochemical, and Histological Study. Heliyon.

[76] Andreeva-Gateva P., Traikov L., Sabit Z., Bakalov D., Tafradjiiska-Hadjiolova R. (2020). Antioxidant Effect of Alpha-Lipoic Acid in 6-Hydroxydopamine Unilateral Intrastriatal Injected Rats. Antioxidants.

[77] Zhang J., Gao Y., Zhang L., Zhang C., Zhao Y., Zhang Y., Li S., Chang C., Zhang X., Yang G. (2022). Alpha-Lipoic Acid Attenuates MPTP/MPP+-Induced Neurotoxicity: Roles of SIRT1-Dependent PGC-1α Signaling Pathways. Neurotox. Res..

[78] Tai S., Zheng Q., Zhai S., Cai T., Xu L., Yang L., Jiao L., Zhang C. (2020). Alpha-Lipoic Acid Mediates Clearance of Iron Accumulation by Regulating Iron Metabolism in a Parkinson’s Disease Model Induced by 6-OHDA. Front. Neurosci..

[79] Zheng Q., Ma P., Yang P., Zhai S., He M., Zhang X., Tu Q., Jiao L., Ye L., Feng Z. (2023). Alpha Lipoic Acid Ameliorates Motor Deficits by Inhibiting Ferroptosis in Parkinson’s Disease. Neurosci. Lett..

[80] de Araújo D.P., De Sousa C.N., Araújo P.V., Menezes C.E., Sousa Rodrigues F.T., Escudeiro S.S., Lima N.B., Patrocínio M.C., Aguiar L.M., Viana G.S. (2013). Behavioral and Neurochemical Effects of Alpha-Lipoic Acid in the Model of Parkinson’s Disease Induced by Unilateral Stereotaxic Injection of 6-OHDA in Rat. Evid.-Based Complement. Altern. Med..

[81] Zhang S.F., Xie C.L., Lin J.Y., Wang M.H., Wang X.J., Liu Z.G. (2018). Lipoic Acid Alleviates L-DOPA-Induced Dyskinesia in 6-OHDA Parkinsonian Rats via Anti-Oxidative Stress. Mol. Med. Rep..

[82] Kulikova O., Troshev D., Berezhnoy D., Stvolinsky S., Timoshina Y., Abaimov D., Muzychuk O., Latanov A., Fedorova T. (2023). Neuroprotective Efficacy of a Nanomicellar Complex of Carnosine and Lipoic Acid in a Rat Model of Rotenone-Induced Parkinson’s Disease. Antioxidants.

[83] Lai Y., Reina-Gonzalez P., Maor G., Miller G.W., Sarkar S. (2025). Biotin Mitigates the Development of Manganese-Induced, Parkinson’s Disease-Related Neurotoxicity in *Drosophila* and Human Neurons. Sci. Signal..

[84] Dovonou A., Bolduc C., Soto Linan V., Gora C., Peralta M.R., Lévesque M. (2023). Animal Models of Parkinson’s Disease: Bridging the Gap between Disease Hallmarks and Research Questions. Transl. Neurodegener..

[85] Jones R.E., Moes N., Zwickey H., Cunningham C.L., Gregory W.L., Oken B. (2008). Treatment of Experimental Autoimmune Encephalomyelitis with Alpha Lipoic Acid and Associative Conditioning. Brain Behav. Immun..

[86] Li B., Gong S., Wu Q., Gao J. (2018). Neuroprotective Effects of α-Lipoic Acid on Long-Term Experimental Autoimmune Encephalomyelitis. Eur. Rev. Med. Pharmacol. Sci..

[87] Chaudhary P., Marracci G., Galipeau D., Pocius E., Morris B., Bourdette D. (2015). Lipoic Acid Reduces Inflammation in a Mouse Focal Cortical Experimental Autoimmune Encephalomyelitis Model. J. Neuroimmunol..

[88] Levy M.J.F., Garcia-Diaz B., Sedel F., Baron-Van Evercooren A., Mozafari S. (2022). High-Dose Pharmaceutical Grade Biotin (MD1003) Accelerates Differentiation of Murine and Grafted Human Oligodendrocyte Progenitor Cells In Vivo. Int. J. Mol. Sci..

[89] Yulug B., Kilic E., Oğuz T., Orhan C., Er B., Tuzcu M., Ozercan I.H., Sahin N., Canpolat S., Komorowski J. (2025). Dose-Dependent Effect of a New Biotin Compound in Hippocampal Remyelination in Rats. Mol. Neurobiol..

[90] Andreassen O.A., Dedeoglu A., Friedlich A., Ferrante K.L., Hughes D., Szabo C., Beal M.F. (2001). Effects of an Inhibitor of Poly(ADP-Ribose) Polymerase, Desmethylselegiline, Trientine, and Lipoic Acid in Transgenic ALS Mice. Exp. Neurol..

[91] Wang T., Cheng J., Wang S., Wang X., Jiang H., Yang Y., Wang Y., Zhang C., Liang W., Feng H. (2018). α-Lipoic Acid Attenuates Oxidative Stress and Neurotoxicity via the ERK/Akt-Dependent Pathway in the Mutant hSOD1-Related *Drosophila* Model and the NSC34 Cell Line of Amyotrophic Lateral Sclerosis. Brain Res. Bull..

[92] Giacobbe A., Hiana J., Wang O., Benatar M., Wicks P., Mascias Cadavid J., ALSUntangled Investigators (2025). ALSUntangled #79: Alpha-Lipoic Acid. Amyotroph. Lateral Scler. Front. Degener..

[93] World Intellectual Property Organization Biotin for Treating Amyotrophic Lateral Sclerosis. WO2016151132A1, 25 March 2016. https://patents.google.com/patent/WO2016151132A1.

[94] Andreassen O.A., Ferrante R.J., Dedeoglu A., Beal M.F. (2001). Lipoic Acid Improves Survival in Transgenic Mouse Models of Huntington’s Disease. NeuroReport.

[95] Mehrotra A., Kanwal A., Banerjee S.K., Sandhir R. (2015). Mitochondrial Modulators in Experimental Huntington’s Disease: Reversal of Mitochondrial Dysfunctions and Cognitive Deficits. Neurobiol. Aging.

[96] Bono-Yagüe J., Gómez-Escribano A.P., Millán J.M., Vázquez-Manrique R.P. (2020). Reactive Species in Huntington Disease: Are They Really the Radicals You Want to Catch?. Antioxidants.

[97] Lim R.G., Al-Dalahmah O., Wu J., Gold M.P., Reidling J.C., Tang G., Adam M., Dansu D.K., Park H.J., Casaccia P. (2022). Huntington Disease Oligodendrocyte Maturation Deficits Revealed by Single-Nucleus RNAseq Are Rescued by Thiamine–Biotin Supplementation. Nat. Commun..

[98] Hager K., Kenklies M., McAfoose J., Engel J., Münch G. (2007). Alpha-Lipoic Acid as a New Treatment Option for Alzheimer’s Disease—A 48 Months Follow-Up Analysis. Neuropsychiatric Disorders: An Integrative Approach.

[99] Shinto L., Quinn J., Montine T., Dodge H.H., Woodward W., Baldauf-Wagner S., Waichunas D., Bumgarner L., Bourdette D., Silbert L. (2014). A Randomized Placebo-Controlled Pilot Trial of Omega-3 Fatty Acids and Alpha Lipoic Acid in Alzheimer’s Disease. J. Alzheimer’s Dis..

[100] Fava A., Pirritano D., Plastino M., Cristiano D., Puccio G., Colica C., Ermio C., De Bartolo M., Mauro G., Bosco D. (2013). The Effect of Lipoic Acid Therapy on Cognitive Functioning in Patients with Alzheimer’s Disease. J. Neurodegener. Dis..

[101] Hager K., Marahrens A., Kenklies M., Riederer P., Münch G. (2001). Alpha-Lipoic Acid as a New Treatment Option for Alzheimer Type Dementia. Arch. Gerontol. Geriatr..

[102] Galasko D.R., Peskind E., Clark C.M., Quinn J.F., Ringman J.M., Jicha G.A., Cotman C., Cottrell B., Montine T.J., Thomas R.G. (2012). Alzheimer’s Disease Cooperative Study. Antioxidants for Alzheimer Disease: A Randomized Clinical Trial with Cerebrospinal Fluid Biomarker Measures. Arch. Neurol..

[103] Kong Y., Zhong J., Wang T., Zhang D. (2025). Association between Dietary Biotin Intake and Dementia Risk, Including Alzheimer’s Disease: A Prospective Study of 122,959 UK Biobank Participants. Mol. Nutr. Food Res..

[104] Cooper J.L. (2008). P3-400: Biotin Deficiency and Abnormal Pantothenic Acid Levels in Dementia. Alzheimer’s Dement..

[105] Rabin M.L., Stevens-Haas C., Havrilla E., Rosenstein A., Toffey B., Devi T., Earnhardt M.C., Kurlan R. (2015). Complementary Therapies for Parkinson’s Disease: What’s Promoted, Rationale, Potential Risks and Benefits. Mov. Disord. Clin. Pract..

[106] Przewodowska D., Marzec W., Madetko N. (2021). Novel Therapies for Parkinsonian Syndromes—Recent Progress and Future Perspectives. Front. Mol. Neurosci..

[107] U.S. National Library of Medicine (2017). Alpha-Lipoic Acid/L-Acetyl Carnitine for Progressive Supranuclear Palsy (NCT01537549). NCT01537549.

[108] Nishiwaki H., Ueyama J., Ito M., Hamaguchi T., Takimoto K., Maeda T., Kashihara K., Tsuboi Y., Mori H., Kurokawa K. (2024). Meta-Analysis of Shotgun Sequencing of Gut Microbiota in Parkinson’s Disease. NPJ Park. Dis..

[109] Rodrigues P., Viero F.T., Trevisan G. (2025). The Impact of α-Lipoic Acid Treatment on Multiple Sclerosis Disability: A Systematic Review and Meta-Analysis of Randomized Controlled Trials. Sclerosis.

[110] Yadav V., Marracci G., Lovera J., Woodward W., Bogardus K., Marquardt W., Shinto L., Morris C., Bourdette D. (2005). Lipoic Acid in Multiple Sclerosis: A Pilot Study. Mult. Scler..

[111] Loy B.D., Fling B.W., Horak F.B., Bourdette D.N., Spain R.I. (2018). Effects of Lipoic Acid on Walking Performance, Gait, and Balance in Secondary Progressive Multiple Sclerosis. Complement. Ther. Med..

[112] Fiedler S.E., Yadav V., Kerns A.R., Tsang C., Markwardt S., Kim E., Spain R., Bourdette D., Salinthone S. (2018). Lipoic Acid Stimulates cAMP Production in Healthy Control and Secondary Progressive MS Subjects. Mol. Neurobiol..

[113] Sedel F., Papeix C., Bellanger A., Touitou V., Lebrun-Frenay C., Galanaud D., Gout O., Lyon-Caen O., Tourbah A., Fontaine B. (2015). High Doses of Biotin in Chronic Progressive Multiple Sclerosis: A Pilot Study. Mult. Scler. Relat. Disord..

[114] Birnbaum G., Stulc J. (2017). High-Dose Biotin as Treatment for Progressive Multiple Sclerosis. Mult. Scler. Relat. Disord..

[115] Tourbah A., Gout O., Vighetto A., Deburghgraeve V., Pelletier J., Papeix C., Lebrun-Frenay C., Labauge P., Brassat D., Toosy A. (2018). MD1003 (High-Dose Pharmaceutical-Grade Biotin) for the Treatment of Chronic Visual Loss Related to Optic Neuritis in Multiple Sclerosis: A Randomized, Double-Blind, Placebo-Controlled Study. CNS Drugs.

[116] Cree B.A.C., Cutter G., Wolinsky J.S., Freedman M.S., Comi G., Giovannoni G., Hartung H.P., Arnold D., Kuhle J., Block V. (2020). Safety and Efficacy of MD1003 (High-Dose Biotin) in Patients with Progressive Multiple Sclerosis (SPI2): A Randomised, Double-Blind, Placebo-Controlled, Phase 3 Trial. Lancet Neurol..

[117] Espiritu A.I., Remalante-Rayco P.P.M. (2021). High-Dose Biotin for Multiple Sclerosis: A Systematic Review and Meta-Analyses of Randomized Controlled Trials. Mult. Scler. Relat. Disord..

[118] Créange A., Hutin E., Sedel F., Le Vigouroux L., Lefaucheur J.P. (2023). High-Dose Pharmaceutical-Grade Biotin in Patients with Demyelinating Neuropathies: A Phase 2b Open-Label, Uncontrolled, Pilot Study. BMC Neurol..

[119] U.S. National Library of Medicine (2020). Explore Neuroprotective Effect of Lipoic Acid in Amyotrophic Lateral Sclerosis (NCT04518540). NCT04518540.

[120] Juntas-Morales R., Pageot N., Bendarraz A., Alphandéry S., Sedel F., Seigle S., Camu W. (2020). High-Dose Pharmaceutical Grade Biotin (MD1003) in Amyotrophic Lateral Sclerosis: A Pilot Study. EClinicalMedicine.

[121] U.S. National Library of Medicine (2018). High Doses of Pharmaceutical-Grade Biotin (MD1003) in Amyotrophic Lateral Sclerosis (NCT03427086). NCT03427086.

[122] Vaddadi K.S., Soosai E., Chiu E., Dingjan P. (2002). A Randomised, Placebo-Controlled, Double-Blind Study of Treatment of Huntington’s Disease with Unsaturated Fatty Acids. NeuroReport.

[123] U.S. National Library of Medicine (2020). Trial of the Combined Use of Thiamine and Biotin in Patients with Huntington’s Disease (HUNTIAM) (NCT04478734). NCT04478734.

[124] Spain R., Powers K., Murchison C., Heriza E., Winges K., Yadav V., Cameron M., Kim E., Horak F., Simon J. (2017). Lipoic Acid in Secondary Progressive MS: A Randomized Controlled Pilot Trial. Neurol. Neuroimmunol. Neuroinflamm..

[125] Teichert J., Hermann R., Ruus P., Preiss R. (2003). Plasma Kinetics, Metabolism, and Urinary Excretion of Alpha-Lipoic Acid following Oral Administration in Healthy Volunteers. J. Clin. Pharmacol..

[126] Hermann R., Mungo J., Cnota P.J., Ziegler D. (2014). Enantiomer-Selective Pharmacokinetics, Oral Bioavailability, and Sex Effects of Various Alpha-Lipoic Acid Dosage Forms. Clin. Pharmacol. Adv. Appl..

[127] Chng H.T., New L.S., Neo A.H., Goh C.W., Browne E.R., Chan E.C. (2009). Distribution Study of Orally Administered Lipoic Acid in Rat Brain Tissues. Brain Res..

[128] Ziegler D., Low P.A., Litchy W.J., Boulton A.J., Vinik A.I., Freeman R., Samigullin R., Tritschler H., Munzel U., Maus J. (2011). Efficacy and Safety of Antioxidant Treatment with α-Lipoic Acid over 4 Years in Diabetic Polyneuropathy: The NATHAN 1 Trial. Diabetes Care.

[129] Ziegler D., Ametov A., Barinov A., Dyck P.J., Gurieva I., Low P.A., Munzel U., Yakhno N., Raz I., Novosadova M. (2006). Oral Treatment with Alpha-Lipoic Acid Improves Symptomatic Diabetic Polyneuropathy: The SYDNEY 2 Trial. Diabetes Care.

[130] National Institute of Diabetes and Digestive and Kidney Diseases (2023). LiverTox: Clinical and Research Information on Drug-Induced Liver Injury—Alpha-Lipoic Acid. https://www.ncbi.nlm.nih.gov/books/NBK591554/.

[131] Turck D., Castenmiller J., de Henauw S., Hirsch-Ernst K.I., Kearney J., Knutsen H.K., Mangelsdorf I., McArdle H.J., Naska A., EFSA Panel on Nutrition, Novel Foods and Food Allergens (NDA) (2021). Scientific Opinion on the Relationship between Intake of Alpha-Lipoic Acid (Thioctic Acid) and the Risk of Insulin Autoimmune Syndrome. EFSA J..

[132] Uchida Y., Ito K., Ohtsuki S., Kubo Y., Suzuki T., Terasaki T. (2015). Major Involvement of Na(+)-Dependent Multivitamin Transporter (SLC5A6/SMVT) in Uptake of Biotin and Pantothenic Acid by Human Brain Capillary Endothelial Cells. J. Neurochem..

[133] Reidling J.C., Nabokina S.M., Said H.M. (2007). Molecular Mechanisms Involved in the Adaptive Regulation of Human Intestinal Biotin Uptake: A Study of the hSMVT System. Am. J. Physiol.-Gastrointest. Liver Physiol..

[134] Li D., Ferguson A., Cervinski M.A., Lynch K.L., Kyle P.B. (2020). AACC Guidance Document on Biotin Interference in Laboratory Tests. J. Appl. Lab. Med..

[135] U.S. Food and Drug Administration (2022). Biotin Interference with Troponin Lab Tests: Assays Subject to Biotin Interference. https://www.fda.gov/medical-devices/in-vitro-diagnostics/biotin-interference-troponin-lab-tests-assays-subject-biotin-interference.

[136] Bongarzone S., Sementa T., Dunn J., Bordoloi J., Sunassee K., Blower P.J., Gee A. (2020). Imaging Biotin Trafficking In Vivo with Positron Emission Tomography. J. Med. Chem..

[137] Office of Dietary Supplements, National Institutes of Health (2022). Biotin—Health Professional Fact Sheet. https://ods.od.nih.gov/factsheets/Biotin-HealthProfessional.

[138] Riverón-Negrete L., Sicilia-Argumedo G., Álvarez-Delgado C., Coballase-Urrutia E., Alcántar-Fernández J., Fernandez-Mejia C. (2016). Dietary Biotin Supplementation Modifies Hepatic Morphology without Changes in Liver Toxicity Markers. BioMed Res. Int..

[139] Ronquillo-Sánchez M.D., Camacho-Carranza R., Fernandez-Mejia C., Hernández-Ojeda S., Elinos-Baez M., Espinosa-Aguirre J.J. (2013). Modulation of the Rat Hepatic Cytochrome P4501A Subfamily Using Biotin Supplementation. BioMed Res. Int..

[140] Lodewyk K., Courtney D.B., Bagnell A., Newton A.S. (2025). Adverse Event Monitoring, Assessment, and Reporting in Nutraceutical and Phytoceutical Trials for Pediatric Neuropsychiatric Conditions: A Systematic Review. J. Psychopharmacol..

[141] Ashrafpour S., Ashrafpour M. (2025). The Double-Edged Sword of Nutraceuticals: Comprehensive Review of Protective Agents and Their Hidden Risks. Front. Nutr..

[142] Timalsina D.R., Abichandani L., Ambad R. (2025). A Review Article on Oxidative Stress Markers F2-Isoprostanes and Presenilin-1 in Alzheimer’s Disease. J. Pharm. Bioallied Sci..

[143] Chen J.J., Kang Y., Gallagher D., Herrmann N., Survilla K., Vieira D., Mah E., Graham S.J., Kiss A., Black S.E. (2025). MRS Demonstrates Elevated Brain Glutathione in Vascular Mild Cognitive Impairment Compared to Cognitively Normal Coronary Artery Disease Controls. Alzheimer’s Dement..

[144] Puliyakkara A., Shirlal A., Pendem S., Priyanka, Kadavigere R., Marike T.S. (2025). Myelin Water Imaging as a Quantitative Diagnostic Tool for Neurodegenerative Diseases: A Systematic Review. Diagnosis.

[145] Banach M., Katsiki N., Latkovskis G., Gaita D., Escobar C., Pella D., Penson P.E., Fogacci F., Reiner Z., Cicero A.F.G. (2024). 2024 Update on Postmarketing Nutrivigilance Safety Profile: A Line of Dietary Food Supplements Containing Red Yeast Rice for Dyslipidemia. Arch. Med. Sci..

